# Environmental selection and evolutionary process jointly shape genomic and functional profiles of mangrove rhizosphere microbiomes

**DOI:** 10.1002/mlf2.12077

**Published:** 2023-09-03

**Authors:** Xiaoli Yu, Qichao Tu, Jihua Liu, Yisheng Peng, Cheng Wang, Fanshu Xiao, Yingli Lian, Xueqin Yang, Ruiwen Hu, Huang Yu, Lu Qian, Daoming Wu, Ziying He, Longfei Shu, Qiang He, Yun Tian, Faming Wang, Shanquan Wang, Bo Wu, Zhijian Huang, Jianguo He, Qingyun Yan, Zhili He

**Affiliations:** ^1^ State Key Laboratory for Biocontrol, Southern Marine Science and Engineering Guangdong Laboratory (Zhuhai), School of Environmental Science and Engineering, Environmental Microbiomics Research Center Sun Yat‐sen University Guangzhou China; ^2^ Institute of Marine Science and Technology Shandong University Qingdao China; ^3^ College of Forestry & Landscape Architecture South China Agricultural University Guangzhou China; ^4^ Southern Marine Science and Engineering Guangdong Laboratory (Zhuhai), School of Marine Science Sun Yat‐sen University Guangzhou China; ^5^ Department of Civil and Environmental Engineering The University of Tennessee Knoxville Tennessee USA; ^6^ Key Laboratory of the Ministry of Education for Coastal and Wetland Ecosystems, School of Life Sciences Xiamen University Xiamen China; ^7^ Xiaoliang Research Station for Tropical Coastal Ecosystems and Key Laboratory of Vegetation Restoration and Management of Degraded Ecosystems, South China Botanical Garden Chinese Academy of Sciences Guangzhou China; ^8^ School of Life Sciences Sun Yat‐sen University Guangzhou China

**Keywords:** average genome size, functional potential, mangrove rhizosphere, metagenome, metagenome‐assembled genome

## Abstract

Mangrove reforestation with introduced species has been an important strategy to restore mangrove ecosystem functioning. However, how such activities affect microbially driven methane (CH_4_), nitrogen (N), and sulfur (S) cycling of rhizosphere microbiomes remains unclear. To understand the effect of environmental selection and the evolutionary process on microbially driven biogeochemical cycles in native and introduced mangrove rhizospheres, we analyzed key genomic and functional profiles of rhizosphere microbiomes from native and introduced mangrove species by metagenome sequencing technologies. Compared with the native mangrove (*Kandelia obovata*, KO), the introduced mangrove (*Sonneratia apetala*, SA) rhizosphere microbiome had significantly (*p* < 0.05) higher average genome size (AGS) (5.8 vs. 5.5 Mb), average 16S ribosomal RNA gene copy number (3.5 vs. 3.1), relative abundances of mobile genetic elements, and functional diversity in terms of the Shannon index (7.88 vs. 7.84) but lower functional potentials involved in CH_4_ cycling (e.g., *mcrABCDG* and *pmoABC*), N_2_ fixation (*nifHDK*), and inorganic S cycling (*dsrAB*, *dsrC*, *dsrMKJOP*, *soxB*, *sqr*, and *fccAB*). Similar results were also observed from the recovered Proteobacterial metagenome‐assembled genomes with a higher AGS and distinct functions in the introduced mangrove rhizosphere. Additionally, salinity and ammonium were identified as the main environmental drivers of functional profiles of mangrove rhizosphere microbiomes through deterministic processes. This study advances our understanding of microbially mediated biogeochemical cycling of CH_4_, N, and S in the mangrove rhizosphere and provides novel insights into the influence of environmental selection and evolutionary processes on ecosystem functions, which has important implications for future mangrove reforestation.

## Impact statement

Understanding the adaptation of microbial communities to changing environments is a critical issue in microbial ecology and evolution, especially reflected in the change of microbial genome sizes and their functional capability of biogeochemical cycles. In this study, we analyzed rhizosphere microbiomes from native and introduced mangrove species and found that the introduced mangrove rhizosphere microbiome tended to adapt to a new or changing environment by recruiting more genetic elements, resulting in a higher average genome size but lower functional potentials of CH_4_ cycling, N_2_ fixation, and inorganic S cycling. Our results revealed the potential mechanism of how native soil microbiomes adapted to the introduced mangrove rhizosphere, advancing our understanding of mangrove rhizosphere microbiomes.

## INTRODUCTION

Microorganisms are ubiquitous and play a profound role in maintaining ecosystem functioning, especially in the biogeochemical cycling of carbon (C), nitrogen (N), and sulfur (S)^1^, ^2^. For instance, microbial communities involved in organic C mineralization, methane (CH_4_) cycling, denitrification, and sulfate reduction could regulate C storage and greenhouse gas emissions (CO_2_, CH_4_, and N_2_O)^3^, ^4^. With recent advances in metagenome sequencing technologies, several studies have explored microbiome functions in a variety of environments, such as ocean^5^, soil^1^, and wetland^6^. Unraveling the functional potential of microbiomes and underlying mechanisms is crucial for mediating and predicting ecosystem functions, especially in response to global change.

Understanding the adaptation of microbial communities to changing environments is a central issue in microbial ecology and evolution^7^. Currently, many studies have observed shifts in both functional and taxonomic compositions of microbial communities in response to environmental changes^8^, ^9^, while few studies explain their underlying mechanisms. The genome size and the rRNA operon copy number are vital metrics for reflecting adaptative and evolutionary processes of microbial communities governed by environmental conditions^9^, ^10^, ^11^. Microbes with low copy numbers can utilize resources more efficiently, whereas those with high copy numbers may have high growth rates^12^, ^13^. The genome size is positively correlated with the 16S rRNA gene copy number and could increase through horizontal gene transfer and duplication or decrease through gene loss^10^, ^12^, ^13^, ^14^, ^15^, ^16^. Both horizontal gene transfer and gene loss are recognized as major driving forces of microbial evolution^10^, ^14^, ^15^, ^16^. Horizontal gene transfer plays a critical role in obtaining novel functionalities to increase the ability of microbes to adapt to a new or changing environment, whereas gene loss of redundant gene duplicates could lead to more efficient functions during the long‐term evolutionary processes^15^, ^16^, ^17^. Generally, microbes that adapt to diverse habitats are considered as generalists with large genomes and versatile metabolic functions, while microbes that adapt to specific habitats as specialists may have small genomes and enrich specific functions with loss of unnecessary genes^18^, ^19^, ^20^. A previous study showed that heated soils lacked genes encoding antimicrobial production and stress responses, leading to smaller genomes relative to those from ambient soils^9^. Also, the experimental evolution of bacteria (e.g., *Methylobacterium extorquens* AM1 and *Salmonella enterica*) could result in genome streamlining or specialization mainly due to the loss of unnecessary genes^16^, ^21^. However, few studies have linked the genome size to ecosystem functions at the community level, impeding our understanding of how environmental selection and evolutionary process influence genomic and functional profiles of microbial communities in the environment.

Mangroves are one of the most productive ecosystems at tropical and subtropical coastlines and possess critical ecological roles such as biogeochemical cycling, C storage, and shoreline protection^22^, ^23^. Mangrove ecosystems are characterized as C‐abundant, N‐limited, and S‐rich environments with specific significance for microbially driven C, N, and S cycling and coupling mechanisms^24^, ^25^. For instance, mangrove sediments with abundant C and S could provide a suitable environment for CH_4_ production and dissimilatory sulfate reduction^4^. As N is limited in mangrove ecosystems, biological N fixation is critical to drive N cycling and mangrove growth^26^. Due to rapid development and urbanization process, the mangrove deforestation rate ranged from 0.16% to 0.39% per annum during 2000–2012 and even higher before 2000^27^, ^28^. Hence, reforestation projects have been carried out worldwide to maintain functions and services of mangroves, and such efforts have improved the conservation of mangroves in recent years^27^, ^28^, ^29^. In China, a native mangrove species *Kandelia obovata* (KO) Sheue, H. Y. Liu and J. Yong and an introduced mangrove species *Sonneratia apetala* (SA) Buch. Ham. were widely afforested in the mangrove reforestation project^28^, ^30^. SA was considered appropriate for mangrove reforestation due to its high growth rate and adaptability^31^; however, our recent results indicated a latent loss of mangrove ecosystem functions, such as decreased C storage and increased CH_4_ emissions^32^, ^33^.

The mangrove rhizosphere is a critical zone that comprises diverse microorganisms directly interacting with plants and is an important microbial hotspot for biogeochemical cycling. Compared with bulk soil, the rhizosphere soil showed distinct microbial communities with strong interactions and selected microbes with specific functional genes^34^, ^35^, ^36^. Plants could produce various chemical compounds, including root exudates, to select specific microbial populations in the rhizosphere, and rhizosphere microbiomes act as linkages of plant–soil–microbe interactions and support plant growth, health, and metabolic functions^35^, ^37^, ^38^. For instance, the mangrove root exudates, such as phenolic acids and fatty acids, played an important role in determining rhizosphere and episphere microbiomes (e.g., denitrifiers and diazotrophs), which are beneficial for N cycling in mangrove ecosystems^35^. Thus, the rhizosphere shows a strong selective pressure for the rhizosphere microbiomes, resulting in the adaptive evolution of microbes. From a wide range of evolutionary perspectives, the native mangrove like KO in China showed a long‐term coevolution with native microbiomes^39^, ^40^, while as an introduced species, SA plantation altered rhizosphere properties such as total N, pH, *Eh*, and salinity due to changes in plant genotype, root exudation, root architecture, and biomass^41^, ^42^, ^43^. Such environmental changes by introduced plants could serve as selective pressures for native microbes, leading to a shift of microbiome diversity, composition, and function in the rhizosphere of introduced plants^41^, ^44^, ^45^. For example, a study of different *Phragmites australis* lineages showed that introduced *P. australis* populations had distinct rhizosphere microbial communities with lower abundances of pathways involved in antimicrobial biosynthesis and degradation^45^. Also, the genotype of red mangrove *Rhizophora mangle* showed a substantial influence on soil bacterial community composition^42^, and such genotypic effects on microbiomes are generally attributed to differences in root exudates, biomass, and C storage capacity^41^, ^43^, ^46^. In our previous study, we found that environmental changes played an essential role in shifting CH_4_ cycling microbial communities and methane emissions^33^. Due to the similar plantation of KO and SA in the Hanjiang River Estuary, this study site provides an excellent research platform to further explore how native soil microbiomes adapt to the introduced mangrove rhizosphere with altered nutrients and environmental conditions.

In this study, we aimed to understand the genomic characteristics and functional potentials of native and introduced mangrove rhizosphere microbiomes and explore their adaptative mechanisms in natural environments. As the native and introduced mangrove rhizosphere microbiome had undertaken different evolutionary processes of genome loss or horizontal gene transfer, we hypothesized that the introduced mangrove rhizosphere microbiome would tend to adapt to a new or changing environment by recruiting more genetic elements (e.g., horizontal gene transfer), resulting in a higher average genome size (AGS) and a higher average copy number (ACN) of microbial communities but lower functional potentials than the native mangrove rhizosphere microbiome. To test this hypothesis, we conducted metagenome sequencing of microbial communities collected from KO and SA rhizospheres and analyzed their AGS, ACN, functional potentials, and recovery of partial or near‐complete metagenome‐assembled genomes (MAGs). This study provides new insights into our understanding of the impact of evolutionary process and environmental selection on mangrove rhizosphere microbiome functions and has important implications to guide future mangrove reforestation.

## RESULTS

### Physiochemical properties of mangrove rhizosphere sediments

We took rhizosphere sediments from the native mangrove species KO and the introduced species SA and measured their physicochemical properties (Figure S1). The results showed that the SA rhizosphere had significantly (Student's *t*‐test, *p* < 0.05) lower salinity (3.5 vs. 12.4 ppt) and lower nutrients, including total carbon (TC) (10.7 vs. 39.8 g kg^−1^), total nitrogen (TN) (1.8 vs. 4.4 g kg^−1^), ammonium (3.4 vs. 7.6 mg kg^−1^), and sulfate (1.92 vs. 3.08 g kg^−1^), but higher pH (7.2 vs. 6.5) compared with the KO rhizosphere. However, nitrate and nitrite concentrations of rhizosphere sediments did not show significant (Student's *t*‐test, *p* > 0.05) differences between the two mangrove species (Table 1).

**Table 1:** Physiochemical properties of mangrove rhizospheres.

Physiochemical property	KO	SA
pH	6.40 ± 0.27	7.11 ± 0.10*
Salinity (ppt)	12.40 ± 1.43*	3.47 ± 0.18
TC (g kg^−1^)	39.78 ± 5.73*	10.66 ± 0.99
TN (g kg^−1^)	4.38 ± 0.32*	1.84 ± 0.31
Nitrate (mg kg^−1^ dry weight)	4.45 ± 0.84	3.56 ± 0.21
Nitrite (mg kg^−1^ dry weight)	52.82 ± 14.96	41.21 ± 9.05
Ammonium (mg kg^−1^ dry weight)	7.55 ± 1.61*	3.42 ± 1.35
Sulfate (g kg^−1^)	3.08 ± 0.72*	1.92 ± 0.11

Data are presented as mean ± SD (standard deviation, *n* = 5). *represents statistically significant difference (*t*‐test, *p* < 0.05) between *Kandelia obovata* (KO) and *Sonneratia apetala* (SA) rhizospheres.

### Genomic and metagenomic characteristics of mangrove rhizosphere microbiomes

**Figure 1:**
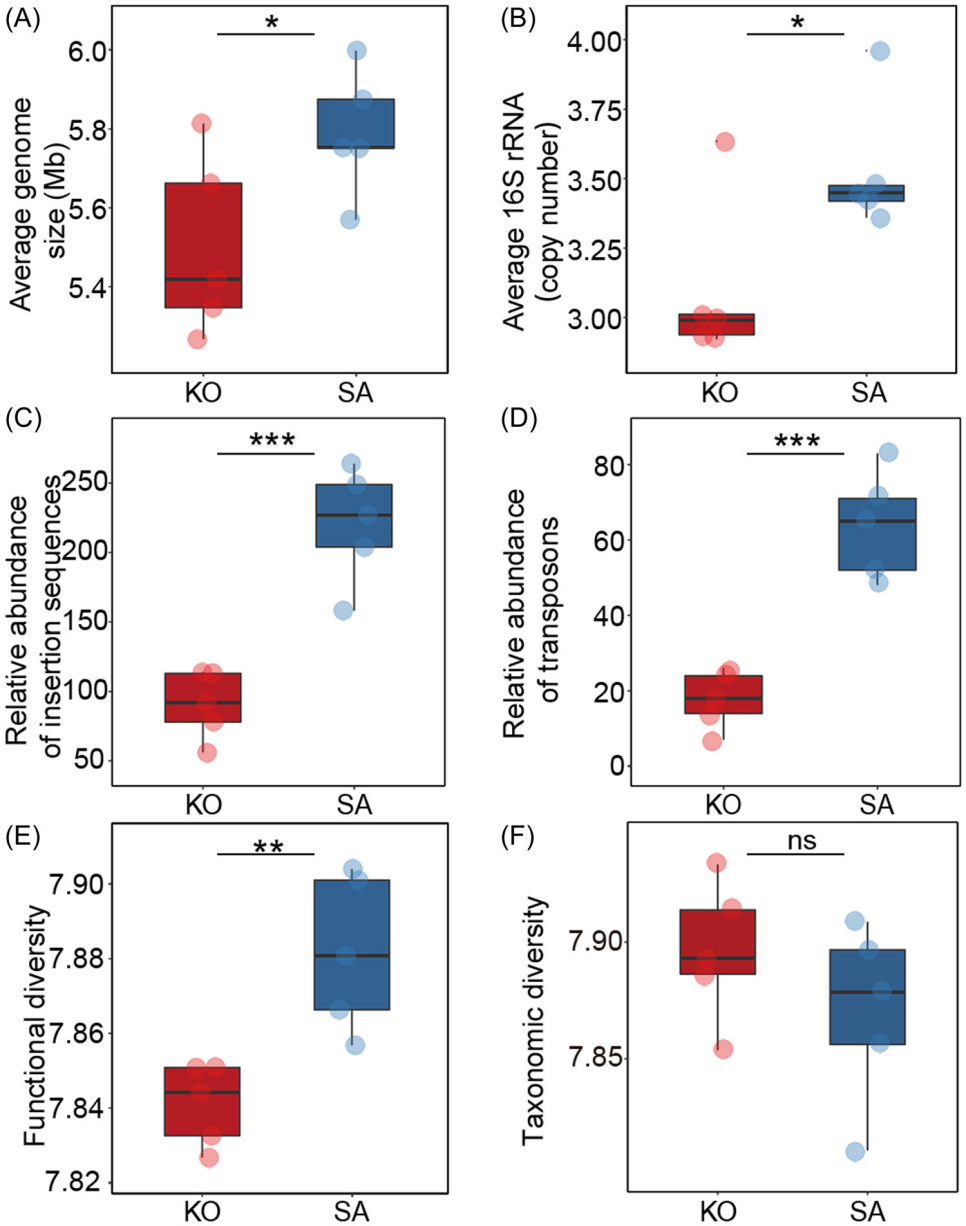
Genomic traits and mobile genetic elements of *Kandelia obovata* (KO) and *Sonneratia apetala* (SA) mangrove rhizosphere microbiomes. (A) Average genome size. (B) Average 16S rRNA copy number. (C) Relative abundances of insertion sequences. (D) Relative abundances of transposons. (E) Functional diversity of rhizosphere microbiomes. (F) Taxonomic diversity of rhizosphere microbiomes. *0.01 < *p* < 0.05; **0.001 < *p* < 0.01; ***p < 0.001; ns, no significant difference.

To explore ecological strategies of mangrove rhizosphere microbiomes, we analyzed the genomic characteristics, such as AGS, ACN, mobile genetic elements (MGEs), and functional and taxonomic diversity, based on metagenome sequencing data. The SA rhizosphere microbiome had significantly (*p* < 0.05) higher AGS (5.8 vs. 5.5 Mb) and ACN (3.5 vs. 3.1) than the KO rhizosphere (Figure 1A,B). We also found that the relative abundance of MGEs, including insertion sequences (220 vs. 91 sequences) and transposons (64 vs. 18 sequences), was significantly higher (*p* < 0.05) in the SA rhizosphere microbiome than in the KO rhizosphere microbiome (Figure 1C,D). Additionally, the functional Shannon index of SA rhizosphere microbiomes was significantly (*p* < 0.05) higher (7.88 vs. 7.84) than that of KO rhizosphere microbiomes (Figure 1E), and the microbiome composition at the gene family level was significantly (*p* < 0.05) different between the two mangrove rhizospheres (Figure S2A). However, no significant (*p* > 0.05) difference in the Shannon index was observed between SA and KO based on taxonomic assignments (Figure 1F).

### Functional potentials of mangrove rhizosphere microbiomes

**Figure 2:**
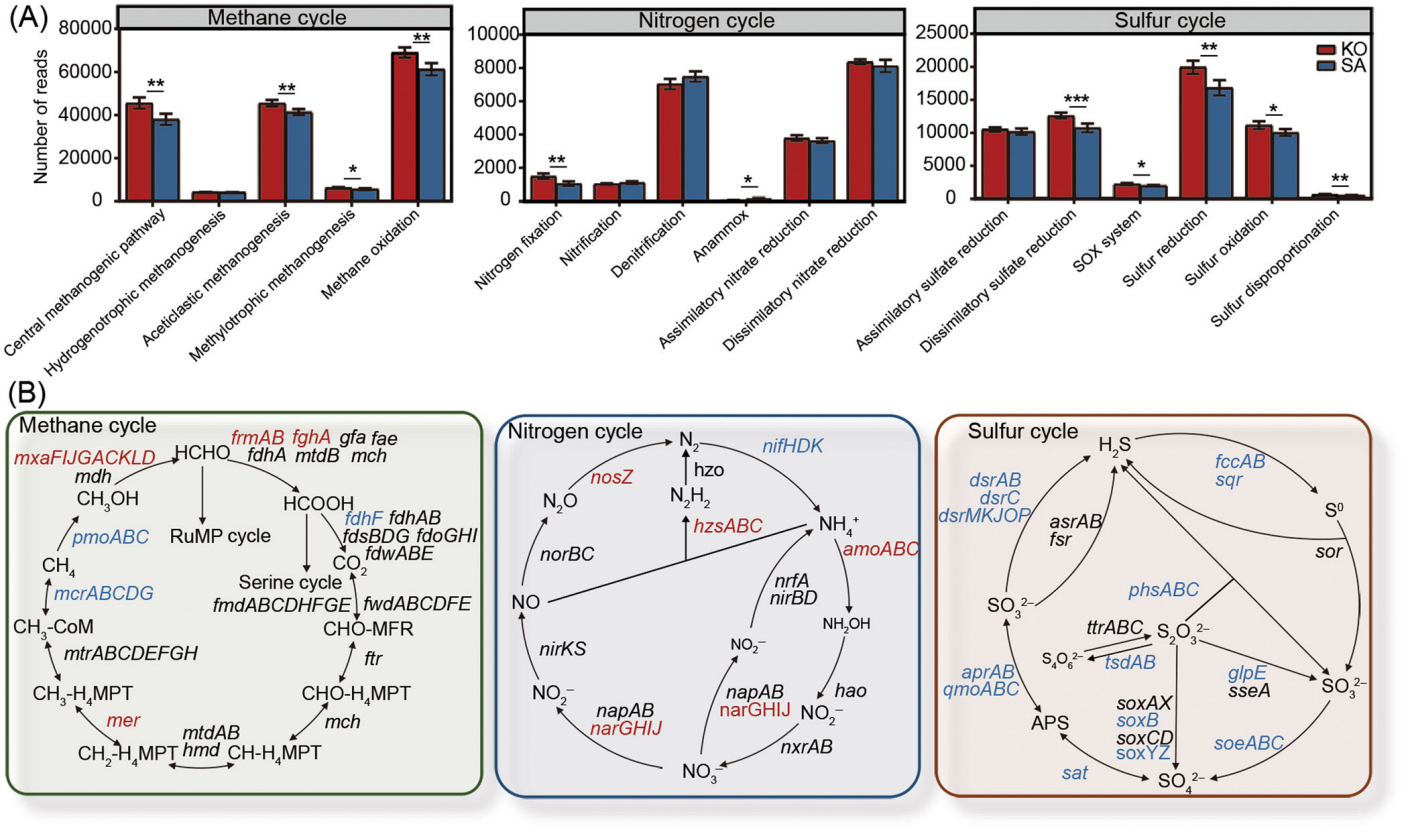
Functional profiles of mangrove rhizosphere microbiomes. (A) Bar plots show the relative abundance of methane, nitrogen, and sulfur cycling pathways between *Kandelia obovata* (KO) and *Sonneratia apetala* (SA) rhizosphere microbiomes. (B) Methane, nitrogen, and sulfur cycling metabolisms with key genes reconstructed from mangrove rhizosphere microbiomes. The red and blue letters represent an increase and a decrease trend of gene abundances in the SA rhizosphere compared with KO, respectively. *0.01 < *p* < 0.05; **0.001 < *p* < 0.01; ***p < 0.001.

The native and introduced mangrove rhizosphere microbiomes also showed differences in functional profiles (Figure 2 and Table S1). Compared with the KO rhizosphere microbiome, the SA rhizosphere microbiome had lower relative abundances of genes involved in carbohydrate metabolism, energy metabolism, nucleotide metabolism, glycan biosynthesis, and metabolism, biosynthesis of other secondary metabolites, and so on (Figure S3). Due to the C‐abundant, N‐limiting, and S‐rich characteristics of mangrove ecosystems, we focused on key gene families involved in CH_4_, N, and S cycling.

For CH_4_ cycling, gene families involved in the central methanogenic pathway, acetoclastic methanogenesis, methylotrophic methanogenesis, and methane oxidation had significantly (Student's *t*‐test, *p* < 0.05) lower relative abundances in the SA rhizosphere than in the KO rhizosphere (Figure 2A). Specifically, the relative abundance of *pmoABC* for the first step of CH_4_ oxidation and *mcrABCDG* for the last step of methanogenesis was significantly (Student's *t*‐test, *p* < 0.05) lower in the SA rhizosphere than in the KO rhizosphere (Figure 2B).

Also, we estimated the abundance of functional genes involved in N cycling and found distinctions in N cycling processes between SA and KO rhizosphere microbiomes (Figure 2). In comparison with the KO rhizosphere microbiome, the SA rhizosphere microbiome had significantly (*p* < 0.05) lower relative abundances of N_2_ fixation but higher relative abundances of anammox (Figure 2A). Specifically, the relative abundance of *nifHDK* responsible for N_2_ fixation was significantly (Student's *t*‐test, *p* < 0.05) lower in the SA rhizosphere than in the KO rhizosphere, consistent with its lower ammonium (Table 1 and Figure 2B). Meanwhile, the relative abundance of *amoABC* for the first step of nitrification was significantly (Student's *t*‐test, *p* < 0.05) higher in the SA rhizosphere microbiome than in the KO rhizosphere microbiome, and similar results were found in *hzsABC* for anammox, *narGHIJ* for nitrate reduction, and *nosZ* for nitrous oxide reduction (Figure 2B).

Additionally, we analyzed gene families involved in S cycling. The results showed that the gene families of dissimilatory sulfate reduction, SOX system, S reduction, S oxidation, and S disproportionation had significantly (Student's *t*‐test, *p* < 0.05) lower relative abundances in the SA rhizosphere microbiome than in the KO rhizosphere microbiome (Figure 2A). First, the SA rhizosphere microbiome showed a significantly (Student's *t*‐test, *p* < 0.05) lower relative abundance of *dsrAB* (the marker of dissimilatory sulfate reduction) than the KO rhizosphere microbiome, and similar results were found for *dsrC* and *dsrMKJOP* (Figure 2B). Also, the gene families involved in thiosulfate oxidation (e.g., *soxB*, *soxYZ*, *glpE*, and *tsdAB*), sulfide oxidation (e.g., *fccAB* and *sqr*), sulfite oxidation (e.g., *soeABC*), and thiosulfate disproportionation (e.g., *phsABC*) showed significantly (Student's *t*‐test, *p* < 0.05) lower relative abundances in the SA rhizosphere microbiome than in the KO rhizosphere microbiome (Figure 2B). Therefore, our results indicated that the SA rhizosphere microbiome had lower functional potentials in CH_4_ cycling, N_2_ fixation, and inorganic S cycling than the KO rhizosphere microbiome.

### Taxonomic composition of mangrove rhizosphere microbiomes

We further analyzed the taxonomic composition of mangrove rhizosphere microbiomes based on Kraken2 assignments. Our results showed that the taxonomic profile of rhizosphere microbiomes had significant (*p* < 0.05) differences at the levels of phylum, class, order, family, genus, and species based on multiple response permutation procedure (MRPP), analysis of similarities (ANOSIM), and permutational multivariate analysis of variance (ADONIS) between KO and SA (Table S1). *Proteobacteria* (34.9%–38.5%) was found to be the dominant phylum in both KO and SA rhizospheres (Figures S4 and S5). Compared with the KO rhizosphere microbiome, the SA rhizosphere microbiome had lower relative abundances of *Burkholderiales*, *Desulfobacterales*, and *Desulfobulbales* but higher abundances of *Rhodobacterales*, both of which were involved in CH_4_/N/S cycling (Figure S6). First, *Desulfobacterales* and *Burkholderiales* associated with methanogenesis (e.g., *mcrABC* and *mtrABC*) were abundant and showed lower relative abundances in the SA rhizosphere (Figure S7). Also, the relative abundance of *Desulfobulbales*, *Campylobacterales*, and *Pseudomonadales* associated with N_2_ fixation was significantly (Student's *t*‐test, *p* < 0.05) lower, while *Burkholderiales* and *Nitrospirales* involved in nitrification and *Rhodobacterales* and *Enterobacterales* involved in denitrification and dissimilatory nitrate reduction were higher in the SA rhizosphere microbiome than in the KO rhizosphere microbiome (Figure S8). In addition, the relative abundance of *Desulfobacterales*, *Burkholderiales*, and *Desulfobulbales*, which are involved in dissimilatory sulfate reduction, was significantly (Student's *t*‐test, *p* < 0.05) lower in the SA rhizosphere microbiome than in the KO rhizosphere microbiome (Figure S9). Consistent with the functional profiles, these results revealed that taxonomic groups involved in CH_4_ cycling, N_2_ fixation, and inorganic S cycling had lower relative abundances, while taxonomic groups in nitrification, denitrification, and anammox showed higher relative abundances in the SA rhizosphere microbiome than in the KO rhizosphere microbiome.

### Recovery of proteobacterial MAGs

**Figure 3:**
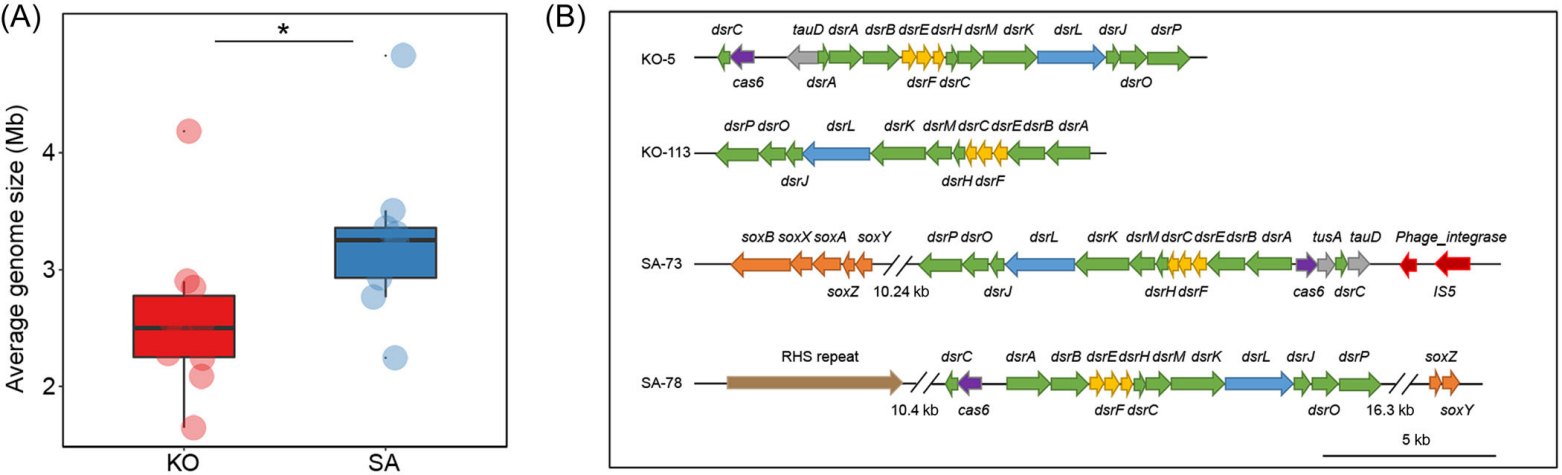
Genomic traits and mobile genetic elements of recovered MAGs. (A) Average genome size of recovered MAGs affiliated with *Proteobacteria*. (B) Organization of dissimilatory sulfur metabolism genes in the recovered MAGs. blue, *dsrL*; brown, RHS repeat; green, *dsrABCMKJOP*; MAGs, metagenome‐assembled genomes; red, mobile genetic elements (insertion sequences and transposons); orange, *soxABXYZ*; purple: *cas6*; yellow, *dsrEFH*.

Assembly and binning analysis led to the recovery of 41 high‐quality (completeness ≥60%, contamination ≤5%) MAGs from mangrove rhizosphere microbiomes (Dataset S1). As *Proteobacteria* was found to be the dominant phylum in the mangrove rhizosphere microbiomes responsible for C, N, and S cycling, we selected 19 proteobacterial MAGs (10 and 9 MAGs from KO and SA, respectively) for further analysis (Table S2). The AGS of proteobacterial MAGs recovered from the SA rhizosphere was significantly (Student's *t*‐test, *p* < 0.05) higher than that from the KO rhizosphere, which is consistent with the read‐based metagenome sequencing data analysis (Figures 1 and 3A). The recovered MAGs were mainly affiliated with *Rhizobiales*, *Burkholderiales*, and *Chromatiales* (Table S2), which were also the dominant orders involved in C, N, and S cycling.

**Figure 4:**
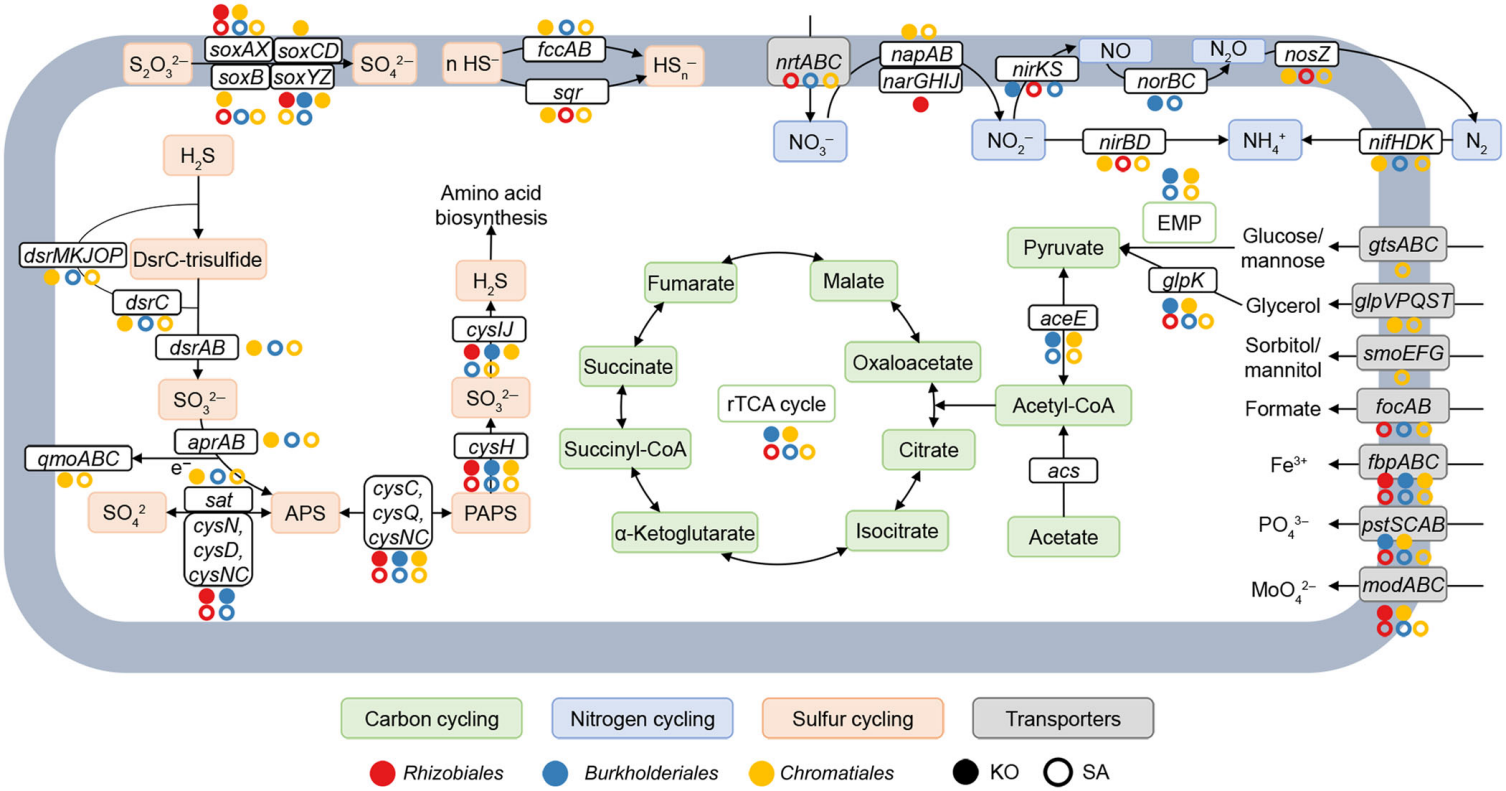
Metabolic pathways of carbon, nitrogen, and sulfur cycling in the three dominant orders (*Rhizobiales*, *Burkholderiales*, and *Chromatiales*) of recovered MAGs. Circles with different colors refer to different orders. The solid and hollow circles indicate the MAGs recovered from *Kandelia obovata* (KO) and *Sonneratia apetala* (SA) rhizospheres, respectively.

To further analyze metabolic pathways of recovered proteobacterial MAGs, we identified their functional genes and annotated their proteins. The results showed that these proteobacterial MAGs from the KO and the SA rhizosphere mainly possessed genetic potentials of dissimilatory sulfate reduction (*dsrAB*, *dsrC*, and *dsrMKJOP*), S oxidation (*soxB*, *soxAX*, and *soxYZ*), N_2_ fixation (*nifHDK*), denitrification (*napAB*, *narGHIJ*, *nirKS*, *norBC*, and *nosZ*), and dissimilatory nitrate reduction (*nirBD*) (Dataset S2). Furthermore, MAGs of the KO rhizosphere had more genes responsible for dissimilatory sulfate reduction (*dsrAB*, *dsrC*, and *dsrMKJOP*) and S oxidation (*sqr* and *fccAB*) (Dataset S2). The MAGs assigned to *Rhizobiales*, *Burkholderiales*, and *Chromatiales* revealed metabolic potentials for S oxidation and nitrate/nitrite reduction, and they showed variations between KO and SA rhizosphere microbiomes (Figure 4). First, the MAGs of SA rhizosphere microbiomes harbored genes encoding various transporters (e.g., *gtsABC*, *smoEFG*, *focAB*, and *nrtABC*), while they were not detected in the same order of MAGs from the KO rhizosphere microbiome. Second, a *Rhizobiales* MAG from the KO rhizosphere (KO‐95) possessed genes encoding nitrate reductase (*narGHIJ*), while a MAG from the SA rhizosphere (SA‐114) possessed genes encoding nitrite reductase (*nirS* and *nirBD*) and nitrous‐oxide reductase (*nosZ*). Third, the MAG of *Burkholderiales* (SA‐73) and the MAGs of *Chromatiales* (KO‐5, KO‐7, KO‐113, SA‐78, and SA‐161) contained genes involved in the dissimilatory sulfate reduction pathway (*dsrABC*, *dsrC*, *dsrEFH*, and *dsrMKJOP*) and SOX systems (*soxAX*, *soxB*, and *soxYZ*) (Figure 4 and Dataset S2). Additionally, the *dsr* gene cluster of SA‐73 had integrase genes and insertion sequences, and SA‐78 contained an RHS repeat (Figure 3B). Therefore, the recovered proteobacterial MAGs from the SA rhizosphere microbiome had a higher AGS and distinct functions compared with the KO mangrove rhizosphere microbiome.

### Potential drivers of mangrove rhizosphere microbiomes

**Figure 5:**
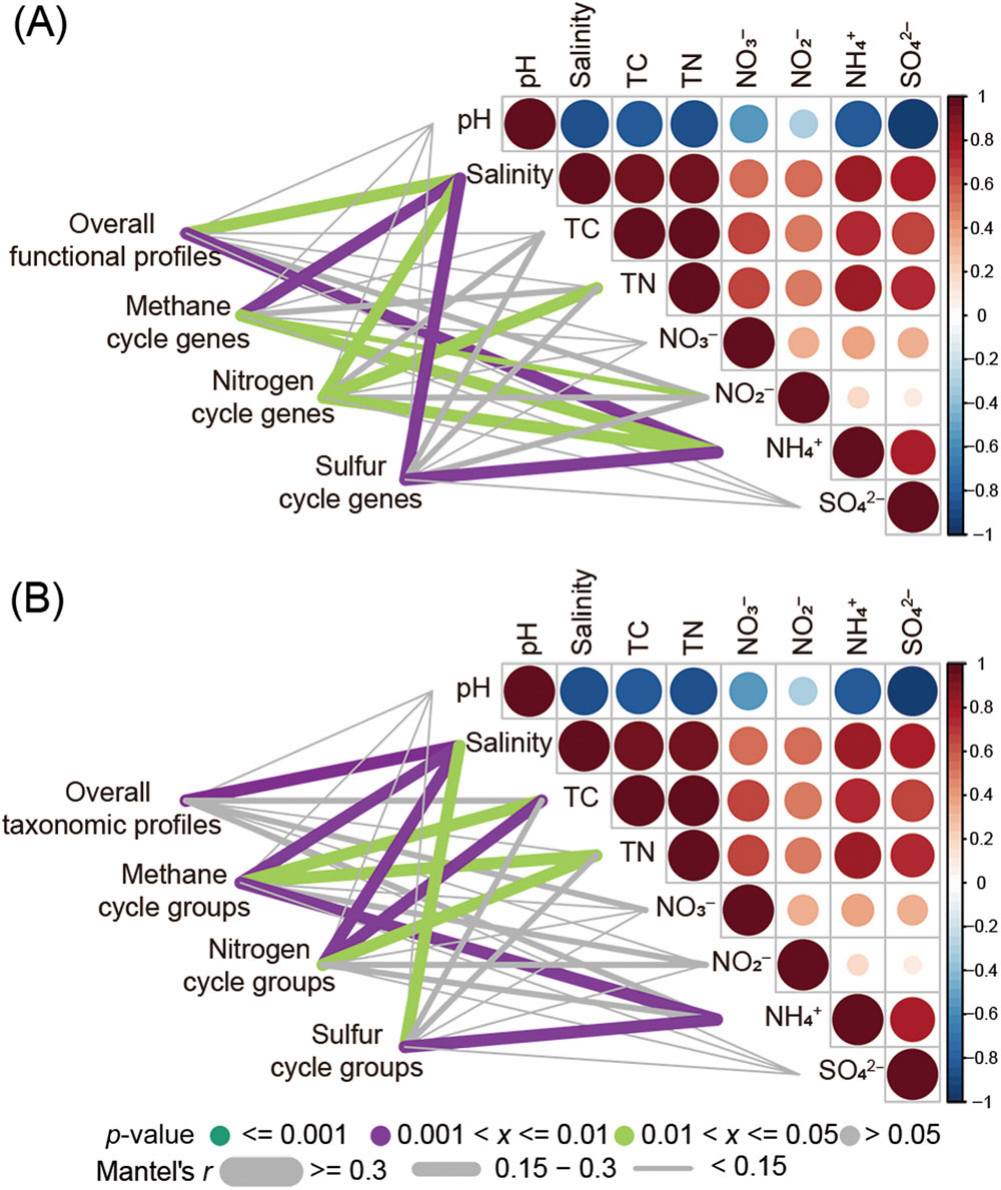
Potential drivers of mangrove rhizosphere microbiomes. The functional (A) and taxonomic (B) composition were related to each environmental factor by partial Mantel tests. Pairwise comparisons of environmental factors were colored by Spearman's correlation coefficients. Edge width represents Mantel's *r* statistic, and edge color represents statistical significance. TC, total carbon; TN, total nitrogen.

We performed the normalized stochasticity ratio (NST), the Mantel test, multiple regression on matrices (MRM), and linear regression analyses to understand potential drivers of mangrove rhizosphere microbiomes. First, the assembly mechanism within functional groups was more deterministic (NST < 50%), while it was more stochastic (NST > 50%) within taxonomic groups (Figure S10). Also, the Mantel test showed that salinity and ammonium were significantly (*p* < 0.05) correlated with the overall and CH_4_/N/S cycling functional profiles of mangrove rhizosphere microbiomes, and the overall and CH_4_/N/S cycling taxonomic profiles were also significantly (*p* < 0.05) correlated with salinity (Figure 5). Further analysis of MRM revealed that salinity made the largest contribution to overall, CH_4_ cycling, and S cycling functional profiles (Table S3). Specifically, salinity and ammonium were significantly (*p* < 0.05) correlated with the genomic features and the relative abundance of specific CH_4_, N, and S cycling pathways (Figures S11–S13). For example, both salinity and ammonium were positively correlated with the relative abundance of CH_4_ cycling pathways (except for hydrogenotrophic methanogenesis), N_2_ fixation, and inorganic S cycling pathways (Figures S11 and S12), while negatively correlated with AGS and the relative abundance of insertion sequences and transposons (Figure S13). Additionally, AGS and the relative abundance of insertion sequences and transposons were negatively correlated with the relative abundance of CH_4_ cycling pathways (except for hydrogenotrophic and methylotrophic methanogenesis), N_2_ fixation, and inorganic S cycling pathways (except for assimilatory sulfate reduction) (Figures S14–S16). Therefore, the results indicated that salinity and ammonium might be the strongest drivers of mangrove rhizosphere microbiomes among the environmental factors examined, and AGS and the relative abundance of MGEs were correlated with functional profiles.

## DISCUSSION

Microbes are crucial to maintain ecosystem functioning, especially in the biogeochemical cycling of C, N, S, phosphorus (P), and metals on the Earth. Mangrove‐associated rhizosphere microbiomes are a global research hotspot for their unique ecosystem characteristics (C‐rich, N‐limited, and S‐rich) and ecosystem functions^24^, ^25^. In this study, we explored the potential impacts of environmental selection and evolutionary process on ecosystem functioning. The results showed that the rhizosphere microbiome of an introduced mangrove had high AGS, ACN, and functional diversity but low functional potentials of CH_4_ cycling, N_2_ fixation, and inorganic S cycling. Such differences were also detected in 19 proteobacterial MAGs recovered from KO and SA rhizosphere microbiomes. Additionally, salinity and ammonium were identified as the main environmental drivers shaping the mangrove rhizosphere microbiome. These results generally support our core hypothesis and provide novel insights into our understanding of the impact of environmental selection and evolutionary process on mangrove ecosystem functioning.

Plants are closely associated with surrounding environments and diverse organisms, and these interactions could play a critical role in plant growth^38^, ^40^. Plant species could influence the soil properties and microbiomes, and the effect of plants on microbiomes is generally attributed to differences in root exudates, biomass, and C storage capacity^41^, ^43^, ^46^. Several studies also reported that coastal ecosystems vegetated with different mangrove species showed substantial variations in sediment properties, including pH, salinity, TC, and TN^32^, ^47^. In this study, we observed different nutrient statuses (TC, TN, ammonium, and sulfate) and environmental conditions (pH and salinity) between native and introduced mangrove rhizospheres, which might be influenced by plant characteristics between native and introduced mangroves^32^, ^48^. For example, the introduced mangrove could accelerate nutrient cycling with higher fine root turnover and decomposition rates, leading to fast growth rates, low carbon storage capacity, and decreased nutrient content in the sediment^32^, ^48^. Root exudates also showed direct impacts on rhizosphere properties (e.g., N and P) and microbial communities^35^, ^46^, ^49^. For instance, the relative abundance of *Hyphomicrobium*, *Nitrospirae*, and diazotrophs was positively correlated with root exudates in the mangrove rhizospheres, revealing the recruitment of microbiome by root exudates^35^. We speculated that the native and introduced mangroves might release distinct root exudates, which shape the rhizosphere microbiomes and select specific microbes^38^, ^49^. Thus, plant characteristics and environmental changes induced by plant characteristics could serve as selective pressures for microbes, leading to environmental selection and evolutionary processes for rhizosphere microbiomes.

Environmental selection is crucial for predicting how ecosystem functions alter in response to a new environment or changing environmental conditions^50^. Previous studies have documented the importance of deterministic selection in structuring microbiomes and revealed that environmental factors had a strong filtering impact^1^, ^5^. Several studies showed that salinity had a significant impact on microbial communities^51^ as salinity could affect microbial metabolic functions by altering enzyme activities and substrate availability^52^. In our previous study, we found that the introduced mangrove with fast nutrient cycling and low C storage capacity had a decreased nutrient content and changed environmental conditions in the sediment, and salinity was the main driver in shaping microbial communities^33^. In this study, our results showed that the functional profile of mangrove rhizosphere microbiomes was mainly shaped by environmental selection, which is consistent with previous studies of global ocean microbiomes and soil microbiomes, showing that functional profiles of microbial communities were shaped by environmental factors, such as dissolved oxygen, salinity, and temperature^2^, ^53^. A possible explanation is that microbial functions are constrained by energetic and nutrient limitations, including the availability of electron donors and acceptors, which were affected by plant characteristics^2^, ^54^. Also, salinity and ammonium were identified as important environmental factors shaping the mangrove rhizosphere microbiome in this study. The introduced mangrove rhizosphere microbiome, characterized by low salinity, showed a low abundance of *dsrAB* but a high abundance of *nosZ*, indicating that high salinity increased the abundance of sulfate reducers and reduced the abundance of *nosZ* ^52^, ^55^. Ammonium is the product of N_2_ fixation and substrate for nitrification, and low ammonium concentrations in the introduced mangrove rhizosphere might be associated with its low relative abundances of functional genes involved in N_2_ fixation (*nifHDK*) and high relative abundances in nitrification (*amoABC*). Furthermore, we found that ammonium was positively correlated with dissimilatory sulfate reduction and S reduction, which could be explained by sulfate reduction coupled with ammonium oxidation under anaerobic conditions^56^. Additionally, environmental selection may impact the genomic features of microbiomes such as AGS, and the native and introduced mangrove rhizosphere environments could select microbial populations with specific AGS and MGEs^9^, ^57^, ^58^. We observed a negative correlation between salinity and AGS or the relative abundance of MGEs, which is consistent with a previous study showing that salinity was negatively correlated with gene richness^57^. These results indicated that environmental factors played important roles in shaping the mangrove rhizosphere microbiomes, and salinity and ammonium affected mangrove rhizosphere microbiomes largely by altering the genomic features and abundance of key functional genes/pathways involved in N and S cycling.

Evolutionary processes, including horizontal gene transfer and gene loss, could shift genomic characteristics (e.g., genome size and rRNA operon copy number) and alter the diversity and function of microbiomes^8^, ^11^, ^14^. Horizontal gene transfer and duplication are considered as main adaptative mechanisms when microbes are exposed to new or changing environments, while the loss of unnecessary or redundant genes could result in more efficient functions during evolutionary processes^16^, ^57^. In this study, we observed high AGS, ACN, and relative abundances of MGEs (e.g., insertion sequences and transposons) in the introduced mangrove rhizosphere microbiome, and their differences may be the result of different evolutionary processes. First, the native mangrove rhizosphere microbiome had a relatively stable environment with a long‐term coevolution with its host, thus resulting in gene loss of redundant gene duplicates with low AGS and high resource use efficiency^39^, ^40^. Second, the introduced mangrove rhizosphere selected its microbiomes capable of adapting to new or changing environments by horizontal gene transfer as it had high relative abundances of MGEs^11^, ^14^. Third, the introduced mangrove rhizosphere might prefer versatile and fast‐growth microbes with large genomes to cope with its fast nutrient cycling and plant tissue degradation^18^, ^39^, ^40^. As the size of microbial genomes corresponds to the number of different genes, genomic features are closely correlated with functional potentials and underlying adaptative mechanisms^10^, ^18^. The introduced mangrove rhizosphere microbiome with higher AGS and ACN could be considered as generalists with multifunctionality, which is also supported by the finding that the introduced mangrove MAGs had more genes encoding transporters^18^, ^19^, ^20^. The high functional diversity of introduced mangrove rhizosphere microbiomes could also be related to more secondary metabolite genes, which could facilitate the plant–microbe interaction and environmental adaptation^59^, ^60^. Evolutionary processes, including gene loss and horizontal gene transfer, are associated with the functional potential of microbiomes, as they altered the number or types of genes in the genome^14^, ^15^. Based on the genome streamlining theory, loss of redundant gene duplicates could lead to more efficient functions^15^, ^16^. The native mangrove rhizosphere microbiome had undergone genome reduction and specialized in efficient biogeochemical cycling with increased abundances of genes and associated microorganisms (e.g., *Desulfobacterales*), which is consistent with the prediction that microbes with smaller genomes are selected for more efficient resource utilization^9^, ^61^. In addition, we found that AGS was negatively correlated with CH_4_ cycling, N_2_ fixation, and inorganic S cycling pathways, which is in agreement with a previous study that specific functions attuned to environments enriched in small genomes^9^. Horizontal gene transfer events, which are often mediated by MGEs, could promote the acquisition of new genes, thereby evolving adaptive traits to cope with changing environments^14^, ^17^. A successful horizontal gene transfer often occurs with operational genes that are expressed at low levels^62^, ^63^. For example, more than 1000 genes have been transferred from bacteria to oxygen‐respiring heterotrophs *Haloarchaea*, including genes responsible for carbon assimilation, respiratory chain complexes, membrane transporters, and cofactor biosynthesis^64^. The ABC transporters may be positively correlated with genome sizes, as large genomes were enriched in regulatory, transport, and secondary metabolism genes^60^. Thus, the introduced mangrove rhizosphere microbiomes can adapt to new or changing environments through horizontal gene transfer with high relative abundances of MGEs, thereby regulating the functional profile by transferring diverse genes that may be associated with regulatory and transport functions^62^, ^63^. In this study, we observed a negative correlation between the relative abundance of MGEs and CH_4_ cycling pathways, N_2_ fixation, and inorganic S cycling pathways, suggesting that introduced mangrove rhizosphere microbiome undergoing horizontal gene transfer may be enriched in specific metabolic functions and thereby compromised in CH_4_ cycling, N_2_ fixation, and inorganic S cycling pathways. However, identifying the function enriched by horizontal gene transfer remains a challenge due to the complexity of microbial communities in natural environments. These results indicated that the difference of genomic characteristics between the native and introduced mangrove rhizosphere microbiomes might be explained by their host intrinsic characteristics and different evolutionary processes of gene loss or horizontal gene transfer, respectively. Evolutionary processes (e.g., gene loss and horizontal gene transfer) are correlated with the functional profile of mangrove rhizosphere microbiomes, while the experimental evidence of horizontal gene transfer (HGT) events using synthetic microbial ecology methods is needed to further understand the interactions between HGT and the functional profile of microbiomes. With the comparison of AGS and ACN of native and introduced mangrove rhizosphere microbiomes conducted at one site only in this study, we acknowledge that further generalization is needed in further studies. For example, the introduced mangrove rhizosphere microbiome in its native habitat should be taken into consideration.

Mangroves are recognized as ecologically and economically important coastal ecosystems, and microbially driven C, N, and S cycling has a critical role in maintaining ecosystem functions^22^, ^26^, ^33^. The functional core microbiota associated with plants is critical for plant growth and health and should show a similar microbiome structure among different plant species^65^. Therefore, functional core microbiota correlated with C, N, and S cycling in the mangrove rhizosphere is believed to be beneficial for maintaining mangrove ecosystem stability, while their different functional potentials between mangrove species may influence mangrove ecosystem versatility. Previous studies revealed that nonnative plants could alter soil nutrients and microbial communities and tend to feed back to benefit themselves over native plants^45^, ^66^. For instance, plant invasion is found to deplete soil N by increasing denitrification and gaseous N losses and potentially causes negative feedback to invasion^67^. We observed distinct functional profiles of the introduced mangrove rhizosphere microbiome, which potentially influence the turnover and output of nutrients. In this study, the decreased N_2_ fixation gene abundances and the increased denitrification gene abundances in the introduced mangrove rhizosphere microbiome revealed a high potential for N loss probably by increased N_2_O emissions^68^. Additionally, sulfate reducers and S oxidizers were the core microbes in coastal sediments, and S oxidizers were reported as the dominant group (e.g., *Gammaproteobacteria*) responsible for C fixation in coastal sediments^69^, ^70^, ^71^. Therefore, the high relative abundance of sulfate reduction and S oxidation genes and associated taxonomic groups in the native mangrove rhizosphere microbiome might be responsible for its high C storage capacity, although the mechanism and contribution of S cycling microbes to C sequestration in mangrove sediments remains to be further studied. Therefore, our results suggested that the introduced mangrove rhizosphere microbiome with reduced functional potentials of N_2_ fixation and inorganic S cycling did not show advantages in nutrient cycling and storage.

**Figure 6:**
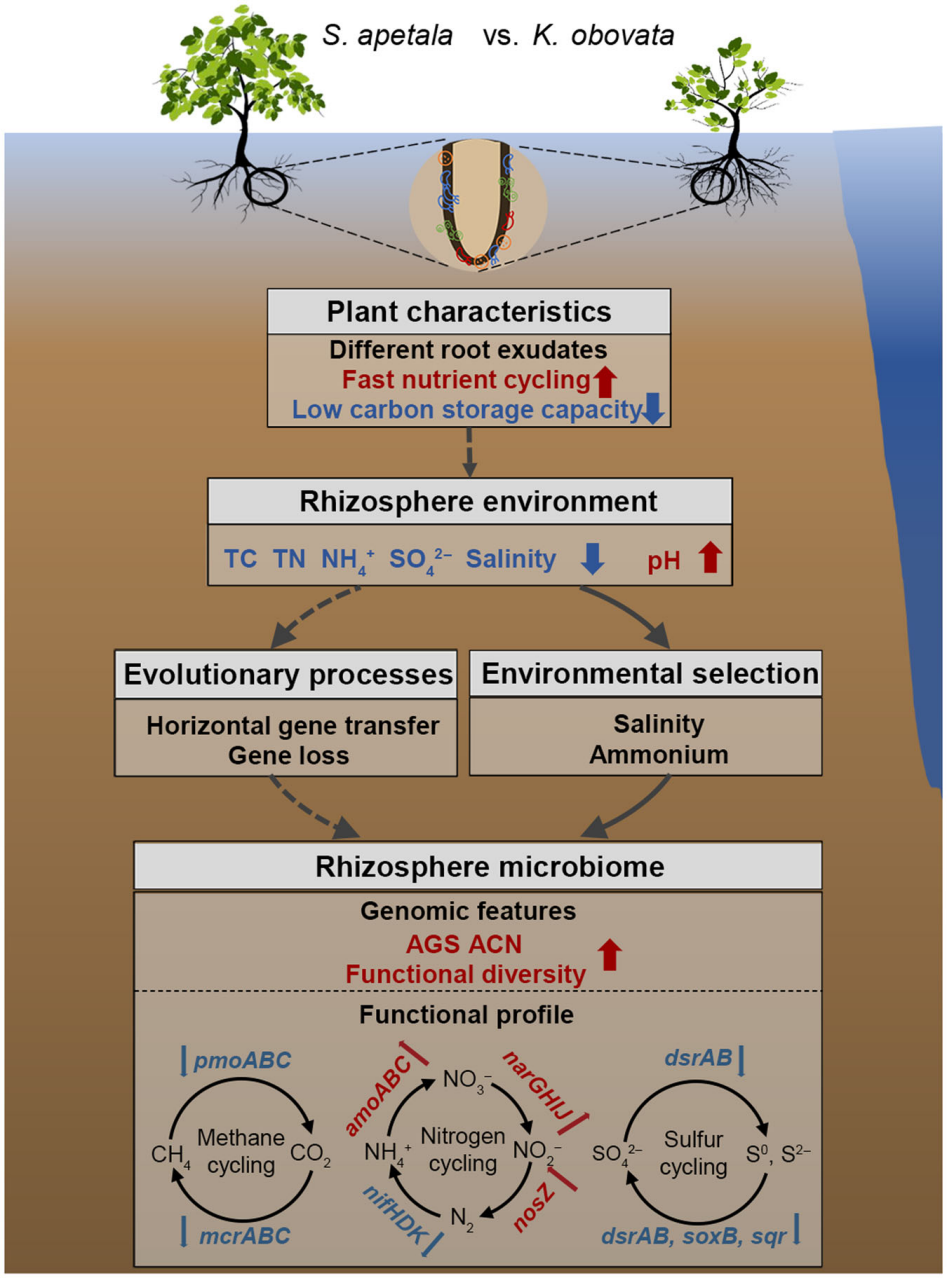
A conceptual model of microbially driven methane, nitrogen, and sulfur cycling as well as their potential drivers in the mangrove rhizosphere. The red and blue arrows represent an increase and a decrease trend of the *Sonneratia apetala* rhizosphere microbiome compared with the *Kandelia obovata* rhizosphere microbiome, respectively. The dashed gray lines indicate possible linkages supported by literature. ACN, average 16S rRNA copy number; AGS, average genome size.

In summary, this study explored the impact of environmental selection and evolutionary process on genomic and functional profiles of mangrove rhizosphere microbiomes (Figure 6). In comparison with the native mangrove, the introduced mangrove with different root exudates, fast nutrient cycling, and low C storage capacity had distinct rhizosphere environments with lower salinity and nutrients (e.g., ammonium, TC, TN, and sulfate). The environmental changes could serve as selective pressures for microbes, leading to environmental selection and evolutionary processes for rhizosphere microbiome. The introduced mangrove rhizosphere microbiome, therefore, showed higher AGS, ACN, and functional diversity than the native one largely due to their differences in adaptative (e.g., horizontal gene transfer) and mangrove–rhizosphere–microbiome co‐evolutionary processes (e.g., gene loss). Such environmental selection and evolutionary processes increased the AGS, ACN, and functional diversity but decreased the functional potential of CH_4_ cycling, N_2_ fixation, and inorganic S cycling in the introduced mangrove rhizosphere microbiome. This study advances our understanding of microbially mediated biogeochemical cycling of CH_4_, N, and S in mangrove ecosystems and provides novel insights into how environmental selection and evolutionary process mediate mangrove rhizosphere microbiome functions, which has important implications for future mangrove reforestation.

## MATERIALS AND METHODS

### Site description and rhizosphere sediment sampling

This mangrove study site is located at the Hanjiang River Estuary (23.27°N, 116.52°E) of Guangdong Province, China, as previously described^32^, ^33^. We collected rhizosphere samples from two mangrove species: the native mangrove KO and the introduced mangrove SA, and they were adjacent with about 1.69 and 1.73 ha, respectively (Figure S1). In 2005, KO and SA seedlings were planted on the same muddy tidal flat with similar environments (4.7% sand, 88.2% silt, and 7.1% clay), tidal levels (1.45–1.55 m), and flooding duration time (10.3–10.6 h) 3 m apart. In July 2018, we selected five individual mangroves for each mangrove species and sampled the rhizosphere sediment (sediments with roots) at a depth of 0–20 cm for each tree. The samples were transported to the laboratory in a portable cooler at 4°C within 24 h. Approximately 1‐mm sediment firmly attached to the root after vigorous shaking was considered as the rhizosphere sediment. The root was placed in phosphate buffer solution (pH 7.0) and shaken for 20 min at 180 rpm. We centrifuged the suspension and collected tight pellets as the rhizosphere sediment^72^. Each rhizosphere sample was divided into two subsamples: one was stored at 4°C for physical and chemical analysis, while the other was kept at −80°C for microbial community DNA extraction.

### Physicochemical property analysis

The rhizosphere sediment samples were dried at 65°C to a constant weight, finely ground, and used for pH, salinity, TC, TN, and sulfate measurements. The pH and salinity were respectively measured using a pH meter (SevenCompact210; Mettler‐Toledo) and a salinity meter (EUTECH SALT6+; Thermo Fisher Scientific) with 2.0 g of dry sediment in a 1:2.5 sediment/water suspension for pH and a 1:5 sediment/water suspension for salinity^32^. Approximately 40 mg of dry rhizosphere sample was used for TC and TN measurement using an elemental analyzer (Vario TOC; Elemental). Dissolved sulfate was extracted with a 1:5 sediment/water suspension, and sulfate concentration was measured by ion chromatography (Dionex ICS‐600; Thermo Fisher Scientific). Fresh rhizosphere samples (2.0 g) were used for nitrate, nitrite, and ammonium extraction with 2 M KCl. Nitrate, nitrite, and ammonium concentrations were measured using the colorimetric method by a multimode microplate reader (Varioskan LUX; Thermo Fisher Scientific).

### DNA extraction and metagenome sequencing

The rhizosphere sediment microbial community DNA was extracted using a classic freeze‐grinding method and purified using a Power Soil DNA Isolation Kit (Mo Bio Laboratories)^73^. DNA quality was measured using a Nanodrop (NanoDrop One; Thermo Fisher Scientific), and the absorbance ratios of 260/280 and 260/230 were about 1.8 and above 1.7, respectively. DNA concentrations were quantified using a fluorescent method (Qubit 4 Fluorometer; Thermo Fisher Scientific). Sequencing libraries were prepared using a VAHTSTM Universal DNA Library Prep Kit for Illumina (Vazyme Biotechnology) following the manufacturer's instructions, and the quality was checked using LabChip GX Touch HT (PerkinElmer). Sequencing was performed using an Illumina NextSeq. 550 platform (2 × 150 paired ends) (Illumina). In total, 208,574,599 raw paired‐end (PE) reads (39–49 million reads per sample) were obtained from the KO rhizosphere and 205,091,594 raw PE reads (35–45 million reads per sample) were obtained from the SA rhizosphere (Table S4).

### Read‐based analysis

Raw paired‐end reads of each sample were trimmed with BBDuk (https://jgi.doe.gov/data-and-tools/bbtools/bb-tools-user-guide/bbduk-guide/) to remove low‐quality reads. The trimmed reads were used to calculate the AGS and the ACN using MicrobeCensus^74^ and Paprica^75^. The AGS was calculated by aligning reads to a set of essential single‐copy genes^74^, and 16S rRNA gene sequences were identified and used for ACN calculation^75^. The trimmed forward and reverse reads were merged using PEAR (option: −p 0.001)^76^. In total, 57,758,963 and 53,635,027 merged reads were retained for the KO and SA rhizosphere, respectively (Table S4). The merged reads were used for both functional and taxonomic annotations. For overall functional annotation, the reads were searched against the KEGG database^77^ by DIAMOND BLASTX alignment (option: ‐e 1e^−5^)^78^, and the reads were further searched against MCycDB for CH_4_ cycling^79^, NCycDB^80^ for N cycling, and SCycDB^81^ for S cycling genes. For overall taxonomic annotation, the merged reads were searched against the Genome Taxonomy Database (version: GTDB‐r207_v2)^82^ using Kraken2's (version: 2.0.8‐beta) k‐mer‐based approach^83^. To obtain the taxonomic profile of CH_4_, N, and S cycling microbiomes, the sequences matched to MCycDB, NCycDB, or SCycDB were extracted using the *seqtk* program^84^, and further annotated using the Kraken2 program^83^. Meanwhile, a random subsampling effort to the minimum sequence number of 8,370,184 was used to normalize both functional and taxonomic annotation, and the number of reads represents the relative abundance of gene families or taxonomic groups. For the MGE analysis, merged reads were aligned to IS sequences from ISfinder^85^, and transposase genes from the MGE database^86^. A read of the best BLASTn hit (e‐value <10^−5^, identity >90%, alignment length >50 bp) was annotated by MGE^87^.

### Metagenome sequence assembly, binning, and annotation

To further explore the genomic characteristics and functional potentials of mangrove rhizosphere microbiomes, the trimmed paired‐end reads of five replicates for each mangrove species were combined and used for genome assembly and binning with the MetaWRAP pipeline^88^. The sequences were assembled into contigs using MEGAHIT (options: −min*k* 21 −max*k* 141 −step 12)^89^, and the assembled contigs were clustered into bins using MetaBAT2^90^ and MaxBin2^91^. To improve the quality of bins, the resulting bins were further improved by the Bin_refinement module and Reassemble_bins module to generate MAGs. The quality of MAGs was evaluated with CheckM^92^, and the MAGs with a high quality (completeness ≥60%, contamination ≤5%) were retained for further analysis^93^. The AGS of MAGs was calculated based on their genome size and completeness. Taxonomic assignments of MAGs were performed using the GTDB‐Tk (version 2.1.0) with its reference data (GTDB‐r207_v2)^82^, and the dominant phyla were selected to compare their genomic features and functions between KO and SA. The encoded proteins of each MAG were predicted with Prodigal (option: ‐p meta)^94^, and predicted amino acid sequences were annotated with eggNOG^95^, KEGG^77^, and Pfam^96^.

### Statistical analysis

All statistical analyses were performed using R (R Foundation for Statistical Computing) or SPSS 22 (SPSS Inc.). The functional or taxonomic diversity of rhizosphere microbiomes was calculated as the Shannon index based on resampled functional and taxonomical profiles using the Galaxy pipeline (http://mem.rcees.ac.cn:8080/). Microbial community dissimilarity was estimated using principal coordinates analysis with Bray–Curtis distances, with statistical significance tested using the MRPP, ANOSIM, and ADONIS. The differential significance of environmental factors, genomic characteristics, diversity indices, genes/pathways, and taxonomic groups between two mangrove species were analyzed by a test of normality (Shapiro–Wilk test) and a variance homogeneity test (Levene's test) and performed using a Student's *t*‐test or nonparametric test (Mann–Whitney *U* test) with SPSS. The assembly mechanism of rhizosphere microbiomes was calculated using an R package “NST,” and the NST was used to assess the ecological stochasticity^97^. The Mantel test and MRM were used to determine the linkage between environmental factors and rhizosphere microbiomes. Linear regression was used to explore the relationship between functional potential and environmental factors or genomic features of rhizosphere microbiomes.

## AUTHOR CONTRIBUTIONS

**Xiaoli Yu**: Conceptualization (supporting); data curation (lead); formal analysis (equal); methodology (lead); visualization (equal); writing—original draft (equal). **Qichao Tu**: Methodology (supporting); resources (lead); writing—review and editing (supporting). **Jihua Liu:** Resources (supporting); writing—review and editing (supporting). **Yisheng Peng**: Conceptualization (supporting); supervision (supporting); writing—review and editing (supporting). **Cheng Wang**: Methodology (supporting); writing—review and editing (supporting). **Fanshu Xiao**: Methodology (supporting). **Yingli Lian**: Data curation (supporting). **Xueqin Yang**: Data curation (supporting). **Ruiwen Hu**: Data curation (supporting). **Huang Yu**: Data curation (supporting). **Lu Qian**: Data curation (supporting). **Daoming Wu**: Writing—review and editing (supporting). **Ziying He**: Writing—review and editing (supporting). **Longfei Shu**: Writing—review and editing (supporting). **Qiang He**: Writing—review and editing (supporting). **Yun Tian**: Writing—review and editing (supporting). **Faming Wang**: Writing—review and editing (supporting). **Shanquan Wang**: Writing—review and editing (supporting). **Bo Wu**: Writing—review and editing (supporting). **Zhijian Huang**: Writing—review and editing (supporting). **Jianguo He**: Writing—review and editing (supporting). **Qingyun Yan**: Supervision (supporting); writing—review and editing (supporting). **Zhili He**: Conceptualization (lead); funding acquisition (equal); supervision (lead); writing—review and editing (lead).

## ETHICS STATEMENT

This study has no animal or human experiments. There are no ethical issues involved.

## CONFLICT OF INTERESTS

The authors declare no conflict of interests.

## Supporting information

Supporting information.

Supporting information.

Supporting information.

## Data Availability

The metagenome dataset has been submitted to the National Omics Data Encyclopedia under an accession number OEX012908 (OES087139‐OES087148).
