# Supplementary material for: Environmental selection and evolutionary process jointly shape genomic and functional profiles of mangrove rhizosphere microbiomes
Source: mLife. 2023 Sep 3;2(3):253–66. doi: 10.1002/mlf2.12077 (PMC10989796; doi:10.1002/mlf2.12077)
Supplement: Supplementary file 3 — Supporting information. [file MLF2-2-253-s002.docx]

**SUPPLEMENTARY INFORMATION**

1. **Supplementary tables**

**Table S1.** Dissimilarity analysis of microbiomes in the mangrove rhizosphere of *K. obovata* and *S. apetala*

**Table S2.** Characteristics of recovered Proteobacterial MAGs

**Table S3.** Relative importance of environmental factors contributing to the functional and taxonomical composition by multiple regression on matrix (MRM) analysis

**Table S4.** Summary of metagenome sequencing data

1. **Supplementary figures**

**Figure S1.** The location of sampling site at the Hanjiang River Estuary, Guangdong Province, China

**Figure S2.** Principal co-ordinates analysis (PCoA) of functional and taxonomical composition of rhizosphere microbiomes of *K. obovata* (KO) and *S. apetala* (SA)

**Figure S3.** The relative abundance of KEGG pathways between *K. obovata* (KO) and *S. apetala* (SA) rhizospheres

**Figure S4.** Taxonomic composition of mangrove rhizosphere microbiomes at the phylum level

**Figure S5.** Taxonomic composition of CH_4_ (A), N (B) and S (C) cycling microbiomes at the phylum level

**Figure S6.** Relative abundances of methane (A), nitrogen (B) and sulfur (C) cycling microbial taxa at the order level

**Figure S7.** Relative abundances of top 15 abundant microbial taxa involved in the central methanogenic pathway (A), hydogenotrophic methanogenesis (B) and aceticlastic methanogenesis (C)

**Figure S8.** Relative abundances of top 15 abundant microbial taxa involved in N_2_ fixation (A), nitrification (B), anammox (C), denitrification (D) and dissimilatory nitrate reduction (E)

**Figure S9.** Relative abundances of top 15 abundant microbial taxa involved in dissimilatory sulfate/sulfur reduction/oxidation (A), SOX system (B), sulfur reduction (C) and sulfur oxidation (D)

**Figure S10.** Assembly mechanisms of functional and taxonomical composition of rhizosphere microbiomes of *K. obovata* (KO) and *S. apetala* (SA)

**Figure S11.** Linear regression analysis of correlations between salinity and relative abundances of CH_4_, N and S cycling pathways

**Figure S12.** Linear regression analysis of correlations between ammonium and relative abundances of CH_4_, N and S cycling pathways

**Figure S13.** Linear regression analysis of correlations between salinity (A) and ammonium (B) with average genome size and relative abundances of insertion sequences and transposons

**Figure S14.** Linear regression analysis of correlations between average genome size and relative abundances of CH_4_, N and S cycling pathways

**Figure S15.** Linear regression analysis of correlations between relative abundances of insertion sequences and CH_4_, N and S cycling pathways

**Figure S16.** Linear regression analysis of correlations between relative abundances of transposons and CH_4_, N and S cycling pathways

**Table S1.** Dissimilarity analysis of microbiomes in the mangrove rhizosphere of *K. obovata* and *S. apetala*.

| Data | **MRPP** | |  | **ANOSIM** | |  | **ADONIS** | |
| --- | --- | --- | --- | --- | --- | --- | --- | --- |
|  | **δ** | ***P*** |  | **R** | ***P*** |  | **F** | ***P*** |
| Kraken2 taxonomy (phylum) | 0.0345 | 0.006 |  | 0.712 | 0.010 |  | 6.6259 | 0.014 |
| Kraken2 taxonomy (class) | 0.0445 | 0.013 |  | 0.716 | 0.009 |  | 7.2498 | 0.026 |
| Kraken2 taxonomy (order) | 0.0521 | 0.010 |  | 0.772 | 0.006 |  | 7.4613 | 0.013 |
| Kraken2 taxonomy (family) | 0.0479 | 0.009 |  | 0.692 | 0.008 |  | 5.6301 | 0.009 |
| Kraken2 taxonomy (genus) | 0.0658 | 0.005 |  | 0.868 | 0.004 |  | 6.8789 | 0.008 |
| Kraken2 taxonomy (species) | 0.0976 | 0.002 |  | 0.880 | 0.005 |  | 5.0477 | 0.006 |
| KEGG ortholog groups | 0.0441 | 0.005 |  | 0.748 | 0.012 |  | 5.9792 | 0.009 |
| Methane cycling (phylum) | 0.0473 | 0.010 |  | 0.748 | 0.013 |  | 7.9171 | 0.011 |
| Methane cycling (class) | 0.0625 | 0.010 |  | 0.728 | 0.015 |  | 8.1341 | 0.004 |
| Methane cycling (order) | 0.0831 | 0.012 |  | 0.752 | 0.005 |  | 6.4537 | 0.012 |
| Methane cycling (family) | 0.0972 | 0.010 |  | 0.912 | 0.007 |  | 6.3916 | 0.014 |
| Methane cycling (genus) | 0.1828 | 0.008 |  | 0.776 | 0.008 |  | 3.0555 | 0.012 |
| Methane cycling (species) | 0.3437 | 0.013 |  | 0.828 | 0.003 |  | 2.1592 | 0.009 |
| Methane cycling gene | 0.0384 | 0.012 |  | 0.68 | 0.016 |  | 8.8512 | 0.008 |
| Nitrogen cycling (phylum) | 0.0535 | 0.009 |  | 0.576 | 0.010 |  | 4.9989 | 0.010 |
| Nitrogen cycling (class) | 0.0704 | 0.015 |  | 0.576 | 0.026 |  | 5.7645 | 0.011 |
| Nitrogen cycling (order) | 0.0990 | 0.011 |  | 0.860 | 0.012 |  | 5.7237 | 0.010 |
| Nitrogen cycling (family) | 0.1298 | 0.007 |  | 0.884 | 0.006 |  | 4.4049 | 0.017 |
| Nitrogen cycling (genus) | 0.2626 | 0.010 |  | 0.772 | 0.009 |  | 2.2720 | 0.009 |
| Nitrogen cycling (species) | 0.5034 | 0.009 |  | 0.680 | 0.005 |  | 1.5974 | 0.006 |
| Nitrogen cycling gene | 0.0378 | 0.011 |  | 0.772 | 0.011 |  | 8.7125 | 0.006 |
| Sulfur cycling (phylum) | 0.0448 | 0.009 |  | 0.700 | 0.008 |  | 6.4433 | 0.012 |
| Sulfur cycling (class) | 0.0588 | 0.002 |  | 0.748 | 0.014 |  | 7.8739 | 0.010 |
| Sulfur cycling (order) | 0.0758 | 0.004 |  | 0.844 | 0.011 |  | 7.1337 | 0.015 |
| Sulfur cycling (family) | 0.0926 | 0.008 |  | 0.872 | 0.009 |  | 5.9891 | 0.015 |
| Sulfur cycling (genus) | 0.1721 | 0.011 |  | 0.872 | 0.005 |  | 3.1824 | 0.007 |
| Sulfur cycling (species) | 0.3472 | 0.004 |  | 0.856 | 0.007 |  | 1.9370 | 0.010 |
| Sulfur cycling gene | 0.0331 | 0.007 |  | 0.712 | 0.014 |  | 8.3229 | 0.012 |

Three non-parametric multivariate analyses (MRPP, ANOSIM and ADONIS) of rhizosphere microbial communities were based on the Bray–Curtis dissimilarity between two mangroves.

**Table S2.** Characteristics of recovered Proteobacterial MAGs.

| Bin | Comple-  teness | Contam-  ination | Quality score | GC | N50 | AGS  (bp) | Contigs | GTDB-Tk taxonomic classification |
| --- | --- | --- | --- | --- | --- | --- | --- | --- |
| KO-95 | 63.38 | 1.021 | 58.275 | 0.625 | 14181 | 1645099 | 198 | d__Bacteria;p__Proteobacteria;c__Alphaproteobacteria;o__Rhizobiales;f__Hyphomicrobiaceae;g__Hyphomicrobium;s__ |
| SA-114 | 90.41 | 3.44 | 73.21 | 0.639 | 8959 | 2764961 | 422 | d__Bacteria;p__Proteobacteria;c__Alphaproteobacteria;o__Rhizobiales;f__Lutibaculaceae |
| KO-21 | 88.7 | 3.556 | 70.92 | 0.644 | 7233 | 2901954 | 513 | d__Bacteria;p__Proteobacteria;c__Alphaproteobacteria;o__Rhodobacterales;f__Rhodobacteraceae;g__HL-12 |
| SA-123 | 82.06 | 1.724 | 73.44 | 0.63 | 13437 | 2930882 | 297 | d__Bacteria;p__Proteobacteria;c__Gammaproteobacteria;o__BM003;f__BM003 |
| SA-163 | 85.19 | 4.579 | 62.295 | 0.614 | 16117 | 3356698 | 377 | d__Bacteria;p__Proteobacteria;c__Gammaproteobacteria;o__BM003;f__BM003;g__BM003 |
| KO-53 | 81.18 | 3.144 | 65.46 | 0.454 | 6754 | 2280408 | 432 | d__Bacteria;p__Proteobacteria;c__Gammaproteobacteria;o__Burkholderiales;f__Nitrosomonadaceae;g__Nitrosomonas |
| SA-100 | 97.48 | 2.073 | 87.115 | 0.455 | 28621 | 3251120 | 197 | d__Bacteria;p__Proteobacteria;c__Gammaproteobacteria;o__Burkholderiales;f__Nitrosomonadaceae;g__Nitrosomonas |
| SA-73 | 86.74 | 3.355 | 69.965 | 0.64 | 31204 | 3506851 | 176 | d__Bacteria;p__Proteobacteria;c__Gammaproteobacteria;o__Burkholderiales;f__Rhodocyclaceae;g__Fen-999 |
| KO-113 | 95.45 | 2.293 | 83.985 | 0.571 | 41520 | 4185942 | 165 | d__Bacteria;p__Proteobacteria;c__Gammaproteobacteria;o__Chromatiales;f__Sedimenticolaceae |
| KO-5 | 90.57 | 2.087 | 80.135 | 0.62 | 9464 | 2477370 | 359 | d__Bacteria;p__Proteobacteria;c__Gammaproteobacteria;o__Chromatiales;f__Sedimenticolaceae |
| KO-7 | 79.31 | 4.672 | 55.95 | 0.584 | 11837 | 2241005 | 384 | d__Bacteria;p__Proteobacteria;c__Gammaproteobacteria;o__Chromatiales;f__Sedimenticolaceae;g__;s__ |
| SA-161 | 92.25 | 2.497 | 79.765 | 0.587 | 31286 | 3309885 | 198 | d__Bacteria;p__Proteobacteria;c__Gammaproteobacteria;o__Chromatiales;f__Sedimenticolaceae |
| SA-78 | 93.3 | 2.944 | 78.58 | 0.561 | 26077 | 4834088 | 304 | d__Bacteria;p__Proteobacteria;c__Gammaproteobacteria;o__Chromatiales;f__Sedimenticolaceae;g__Thiodiazotropha |
| SA-171 | 63.1 | 4.655 | 39.825 | 0.433 | 4431 | 3131987 | 817 | d__Bacteria;p__Proteobacteria;c__Gammaproteobacteria;o__Enterobacterales;f__Vibrionaceae;g__Vibrio;s__ |
| KO-80 | 94.57 | 2.298 | 83.08 | 0.493 | 75177 | 2850300 | 97 | d__Bacteria;p__Proteobacteria;c__Gammaproteobacteria;o__SZUA-229;f__SZUA-229 |
| SA-51 | 73.39 | 2.406 | 61.36 | 0.501 | 8030 | 2244977 | 389 | d__Bacteria;p__Proteobacteria;c__Gammaproteobacteria;o__SZUA-229;f__SZUA-229 |
| KO-111 | 65.51 | 0 | 65.51 | 0.62 | 5483 | 2085452 | 456 | d__Bacteria;p__Proteobacteria;c__Gammaproteobacteria;o__Thiohalobacterales;f__Thiohalobacteraceae;g__UBA9214 |
| KO-23 | 89.7 | 2.23 | 78.55 | 0.534 | 48789 | 2559930 | 90 | d__Bacteria;p__Proteobacteria;c__Gammaproteobacteria;o__Thiohalomonadales;f__SZUA-152;g__SZUA-152 |
| KO-156 | 66.37 | 0.862 | 62.06 | 0.633 | 6944 | 2523193 | 599 | d__Bacteria;p__Proteobacteria;c__Gammaproteobacteria;o__Xanthomonadales;f__SZUA-36 |

**Table S3.** Relative importance of environmental factors contributing to the functional and taxonomical composition by multiple regression on matrix (MRM) analysis.

|  | Overall  functional profiles  r^2^=0.655, *P*=0.008 | | Methane  cycle genes  r^2^=0632, *P*=0.012 | | Nitrogen  cycle genes  r^2^=0.581, *P*=0.017 | | Sulfur  cycle genes  r^2^=0.708, *P*=0.007 | | Overall  taxonomic profiles  r^2^=0.732, *P*=0.005 | | Methane  cycle groups  r^2^=0.164, *P*=0.671 | | Nitrogen  cycle groups  r^2^=0.203 *P*=0.610 | | Sulfur  cycle groups  r^2^=0.159, *P*=0.621 | |
| --- | --- | --- | --- | --- | --- | --- | --- | --- | --- | --- | --- | --- | --- | --- | --- | --- |
|  | Coefficient | *P* | Coefficient | *P* | Coefficient | *P* | Coefficient | *P* | Coefficient | *P* | Coefficient | *P* | Coefficient | *P* | Coefficient | *P* |
| pH | -0.015 | 0.332 | -0.033 | 0.107 | -0.015 | 0.484 | -0.025 | 0.191 | -0.003 | 0.852 | -0.005 | 0.891 | -0.028 | 0.546 | -0.046 | 0.237 |
| Ln(Salinity) | **0.031** | **0.010** | **0.044** | **0.007** | 0.026 | 0.066 | **0.046** | **0.003** | 0.005 | 0.746 | 0.006 | 0.853 | -0.034 | 0.296 | 0.003 | 0.907 |
| Ln(TC) | -0.012 | 0.387 | -0.026 | 0.151 | -0.013 | 0.47 | -0.021 | 0.192 | 0.026 | 0.133 | -0.031 | 0.406 | 0.056 | 0.172 | 0.004 | 0.905 |
| Ln(TN) | -0.010 | 0.453 | 0.005 | 0.786 | 0.009 | 0.654 | -0.004 | 0.827 | -0.018 | 0.376 | 0.022 | 0.582 | -0.035 | 0.380 | 0.021 | 0.580 |
| Ln(NO_3_^-^) | -0.002 | 0.865 | -0.014 | 0.445 | -0.006 | 0.706 | -0.018 | 0.228 | -0.018 | 0.306 | 0.008 | 0.861 | -0.014 | 0.747 | 0.027 | 0.474 |
| Ln(NO_2_^-^) | 0.002 | 0.681 | 0.006 | 0.484 | 0.004 | 0.582 | 0.002 | 0.802 | -0.014 | 0.098 | -0.021 | 0.229 | 0.001 | 0.972 | 0.0004 | 0.988 |
| Ln(NH_4_^+^) | **0.015** | **0.012** | 0.014 | 0.069 | 0.012 | 0.132 | **0.015** | **0.043** | **0.025** | **0.001** | 0.016 | 0.331 | -0.003 | 0.849 | -0.009 | 0.512 |
| Ln(SO_4_^2-^) | 0.013 | 0.464 | 0.029 | 0.194 | 0.010 | 0.631 | 0.018 | 0.354 | 0.004 | 0.838 | 0.013 | 0.778 | 0.053 | 0.296 | 0.029 | 0.482 |

**Table S4.** Summary of metagenome sequencing data.

|  | Raw PE reads | Raw data size (Gb) | Q20% | Q30% | GC% | Clean PE reads | Merged reads |
| --- | --- | --- | --- | --- | --- | --- | --- |
| KO1 | 39,400,737 | 11.82 | 99.57 | 87.28 | 57.17 | 34,961,309 | 13,209,811 |
| KO2 | 39,715,696 | 11.91 | 99.58 | 86.85 | 56.01 | 35,021,314 | 11,319,315 |
| KO3 | 49,452,093 | 14.84 | 99.58 | 84.30 | 56.33 | 41,909,520 | 11,646,683 |
| KO4 | 39,346,440 | 11.80 | 99.54 | 86.34 | 55.90 | 34,497,966 | 9,409,374 |
| KO5 | 40,659,633 | 12.20 | 99.54 | 86.91 | 55.89 | 35,921,633 | 12,173,780 |
| SA1 | 35,747,748 | 10.72 | 99.54 | 86.52 | 56.56 | 31,378,184 | 9,768,870 |
| SA2 | 44,258,041 | 13.28 | 99.50 | 85.02 | 56.78 | 38,226,608 | 12,725,476 |
| SA3 | 41,619,742 | 12.49 | 99.53 | 86.16 | 56.48 | 36,222,190 | 9,731,628 |
| SA4 | 45,192,675 | 13.56 | 99.54 | 86.37 | 55.76 | 39,490,252 | 13,038,869 |
| SA5 | 38,273,343 | 11.48 | 99.53 | 86.47 | 56.63 | 33,435,823 | 8,370,184 |


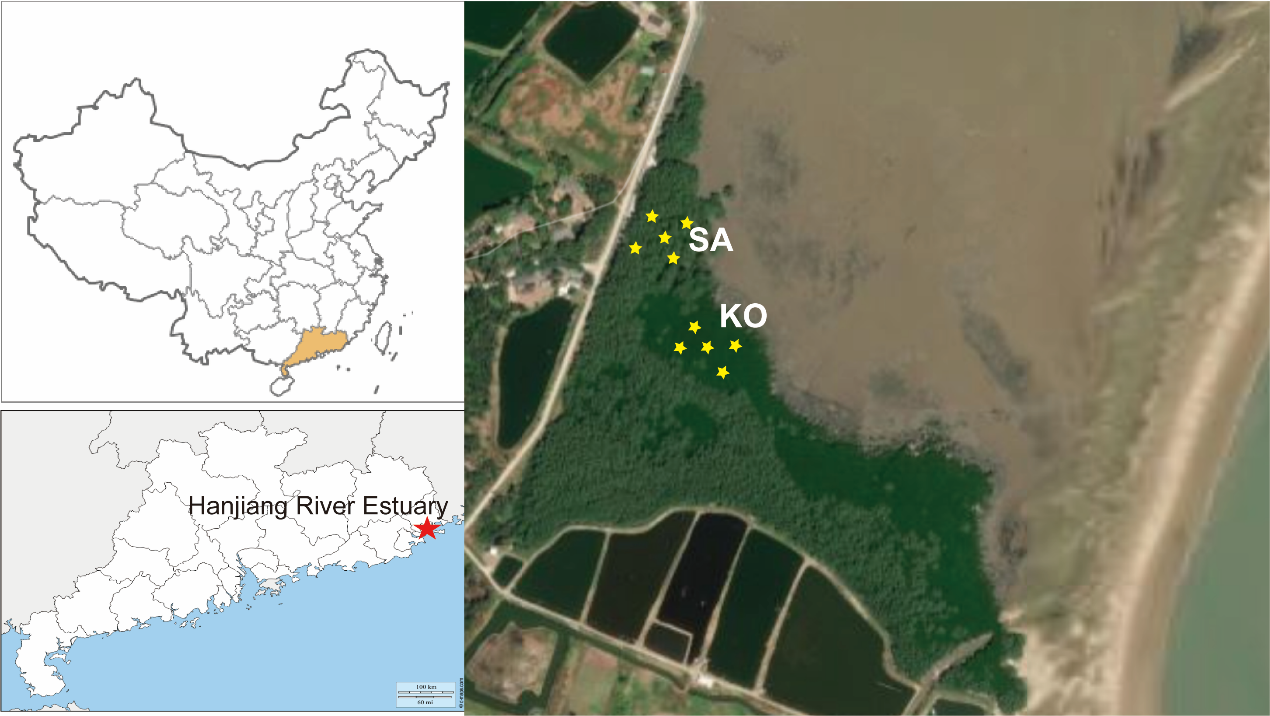
**Figure S1.** The location of sampling site at the Hanjiang River Estuary, Guangdong Province, China.


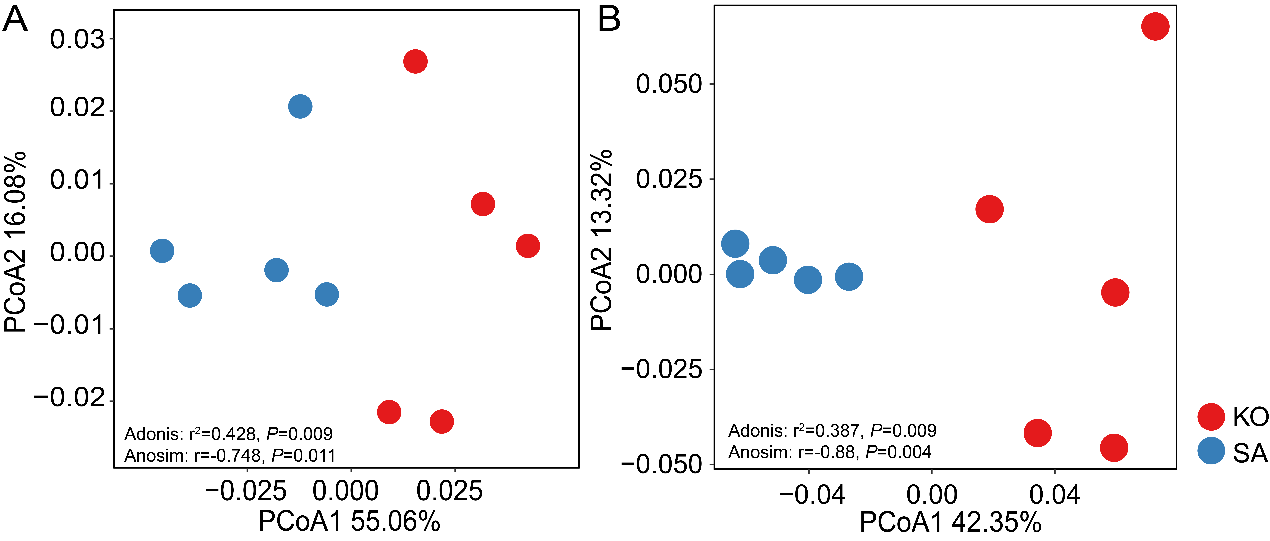


**Figure S2.** Principal co-ordinates analysis (PCoA) of functional and taxonomical composition of rhizosphere microbiomes of *K. obovata* (KO) and *S. apetala* (SA). (A) PCoA plot of functional composition based on KEGG annotations; (B) PCoA plot of taxonomical composition based on Kraken2 classification at the species level.


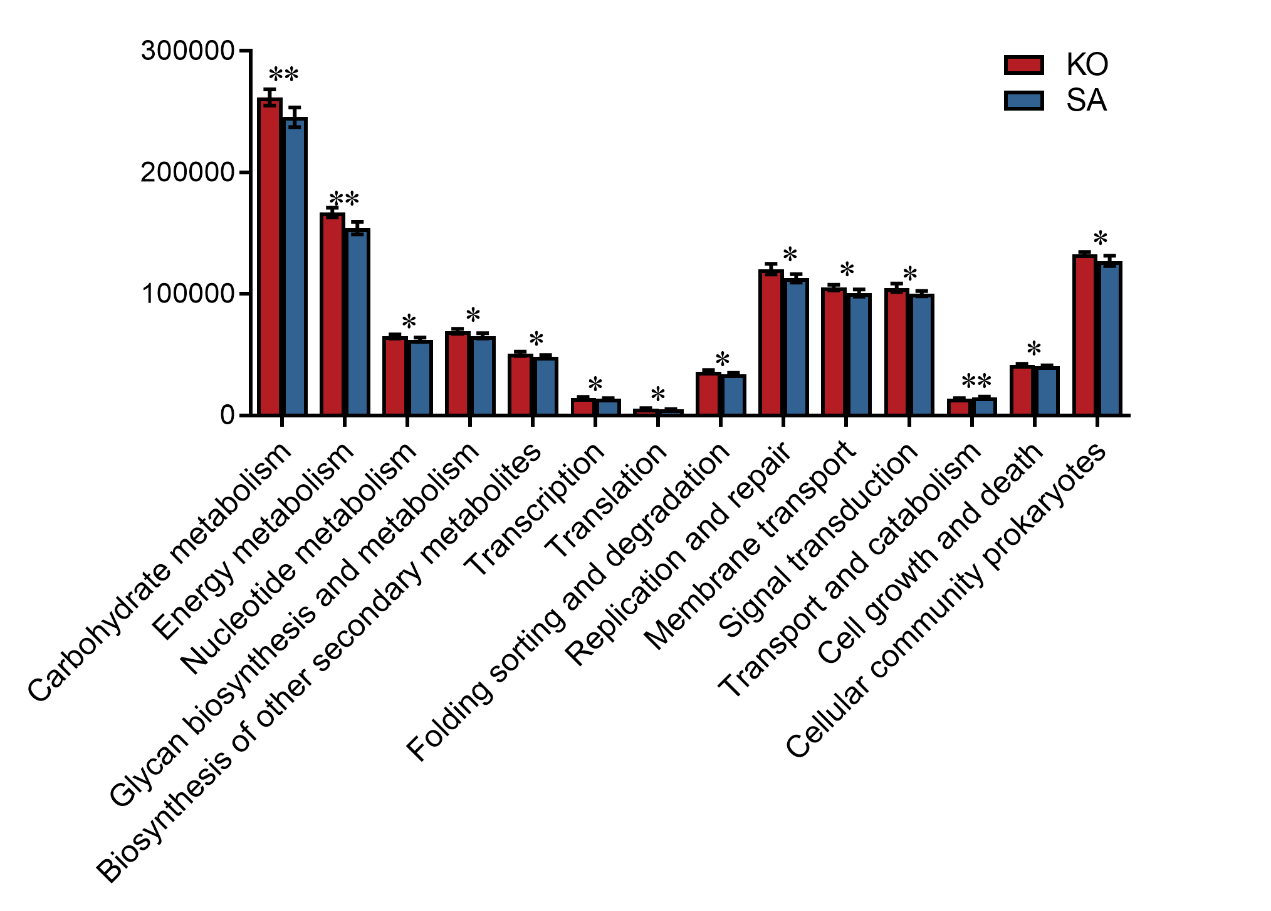


**Figure S3** The relative abundance of KEGG pathways between *K**.* *obovata* (KO) and *S. apetala* (SA) rhizospheres. *: 0.01<*P*<0.05; **: 0.001<*P*<0.01; ***: *P*<0.001.


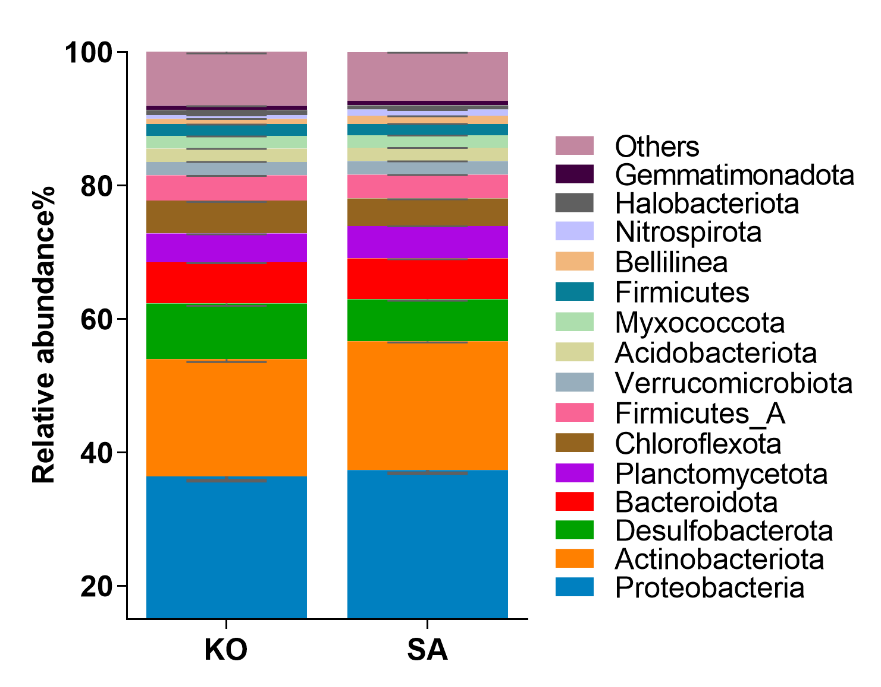


**Figure S4.** Taxonomic composition of mangrove rhizosphere microbiomes at the phylum level. Different colors refer to different phyla. KO: *K. obovata*; SA: *S. apetala*.


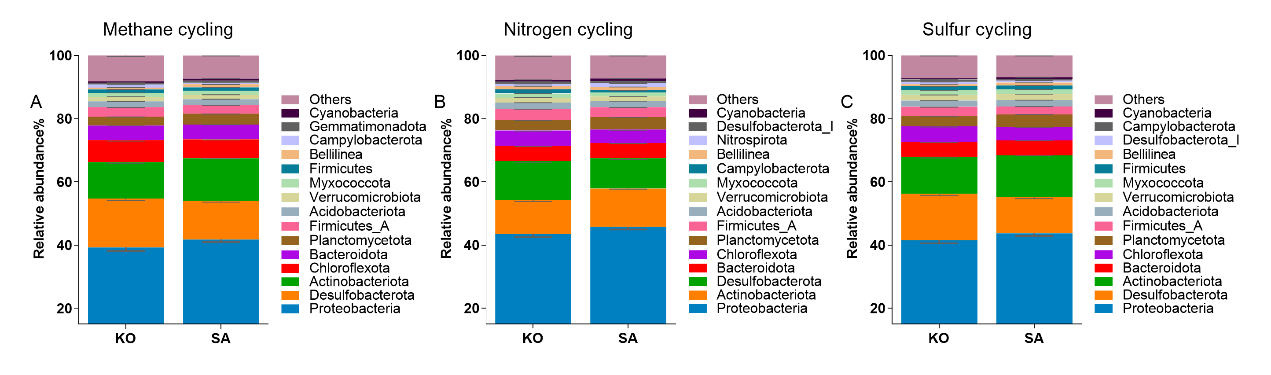


**Figure S5.** Taxonomic composition of CH_4_ (A), N (B) and S (C) cycling microbiomes at the phylum level. Different colors refer to different phyla. KO: *K. obovata*; SA: *S. apetala*.


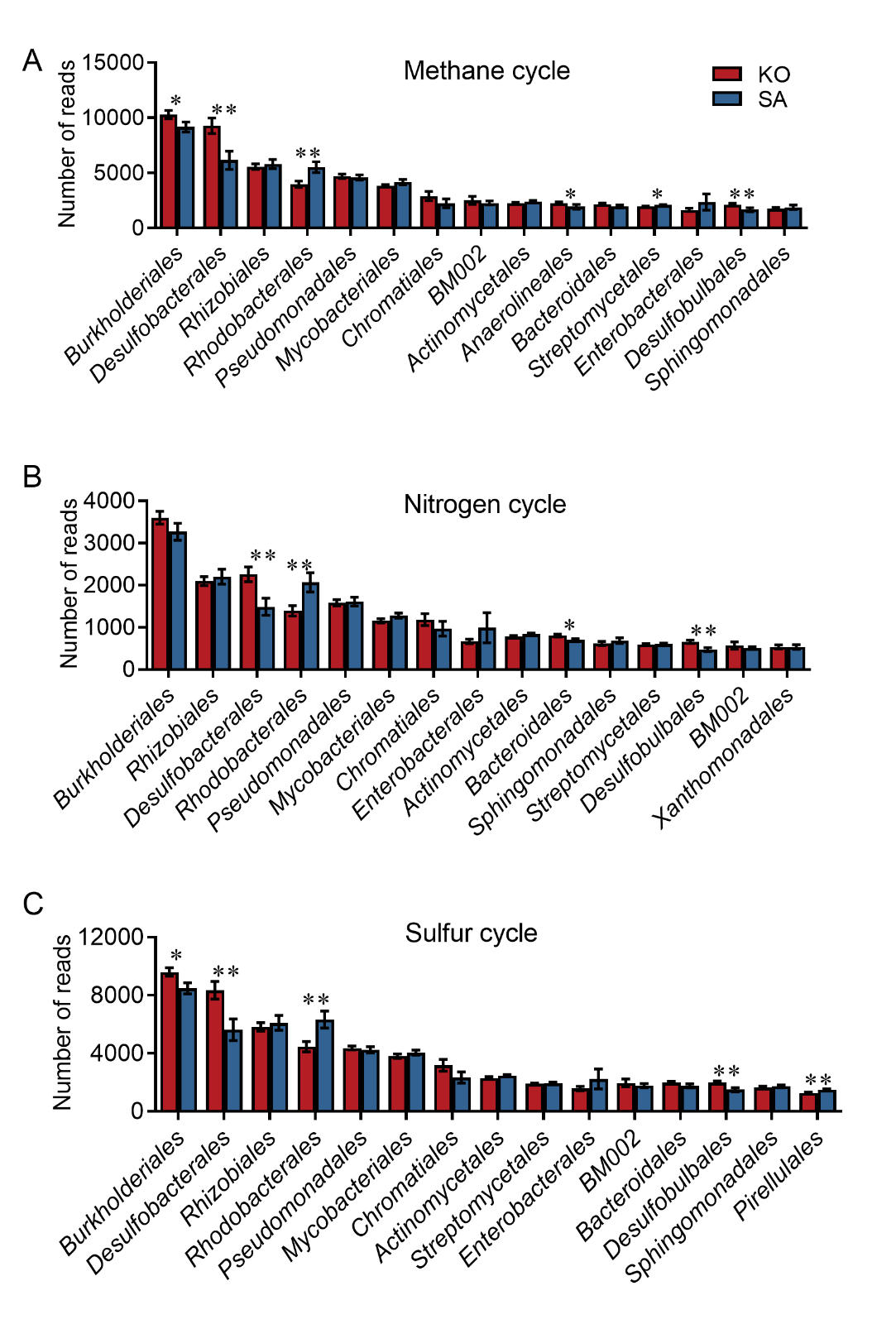


**Figure S6.** Relative abundances of methane (A), nitrogen (B) and sulfur (C) cycling microbial taxa at the order level. Bars represent the normalized number of reads for each order at a random subsampling of 8,370,184 sequences per sample. Data are presented as mean ± SD (standard deviation, n = 5). *: 0.01<*P*<0.05; **: 0.001<*P*<0.01; ***: *P*<0.001. KO: *K. obovata*; SA: *S. apetala*.


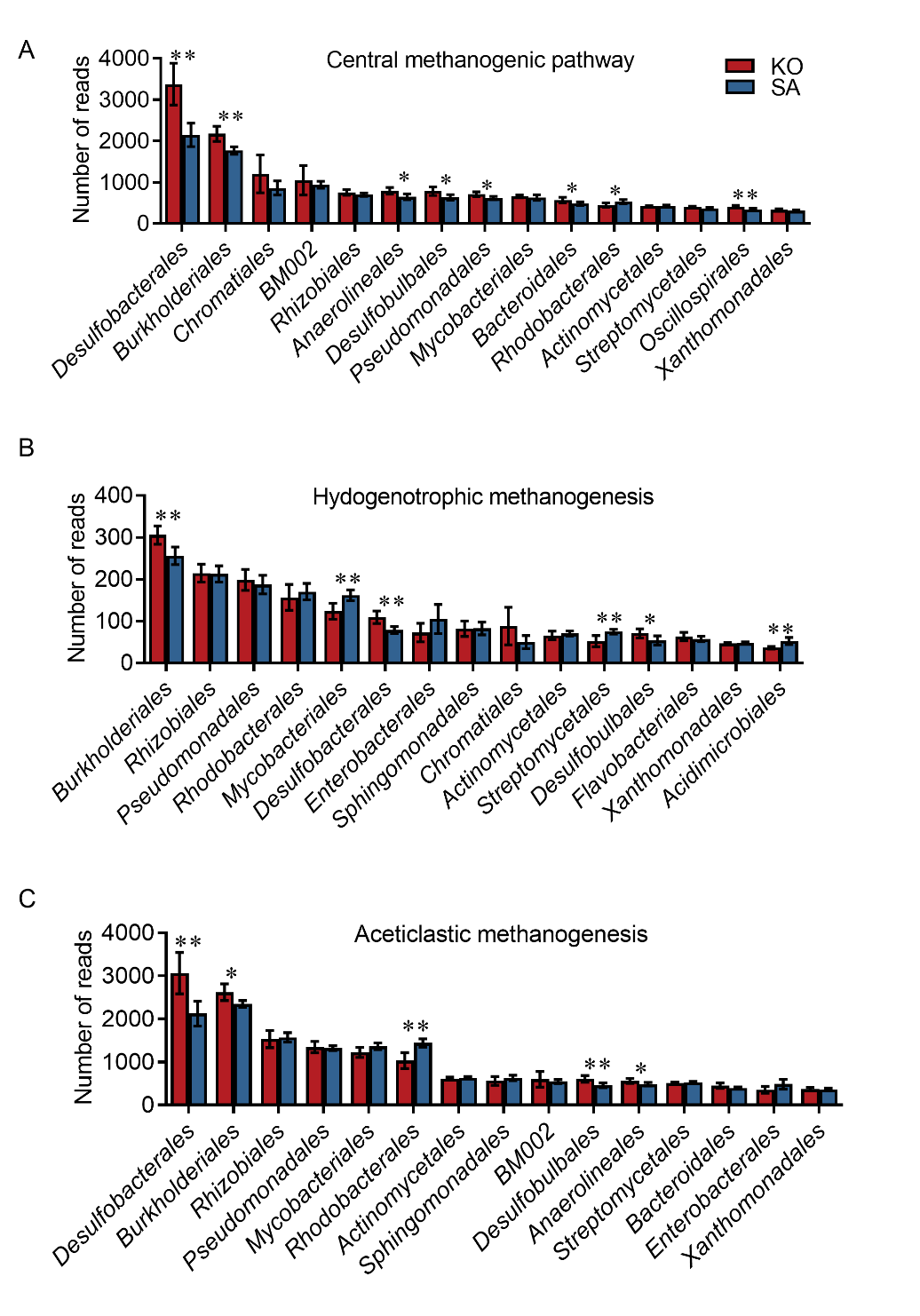


**Figure S7.** Relative abundances of top 15 abundant microbial taxa involved in the central methanogenic pathway (A), hydogenotrophic methanogenesis (B) and aceticlastic methanogenesis (C). Bars represent the normalized number of reads for each order at a random subsampling of 8,370,184 sequences per sample. Data are presented as mean ± SD (standard deviation, n = 5). *: 0.01<*P*<0.05; **: 0.001<*P*<0.01; ***: *P*<0.001. KO: *K. obovata*; SA: *S. apetala*.


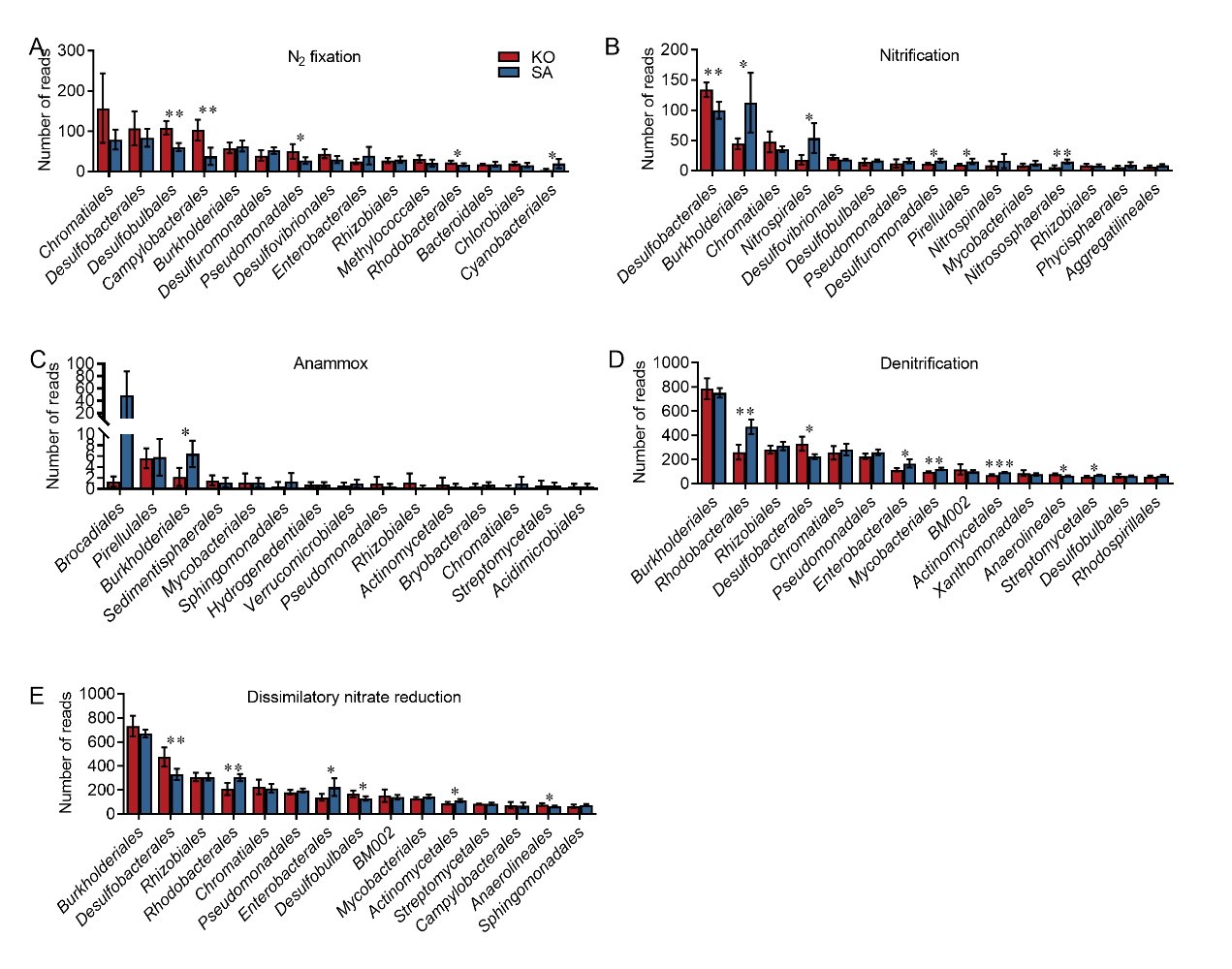


**Figure S8.** Relative abundances of top 15 abundant microbial taxa involved in N_2_ fixation (A), nitrification (B), anammox (C), denitrification (D) and dissimilatory nitrate reduction (E). Bars represent the normalized number of reads for each order at a random subsampling of 8,370,184 sequences per sample. Data are presented as mean ± SD (standard deviation, n = 5). *: 0.01<*P*<0.05; **: 0.001<*P*<0.01; ***: *P*<0.001. KO: *K. obovata*; SA: *S. apetala.*


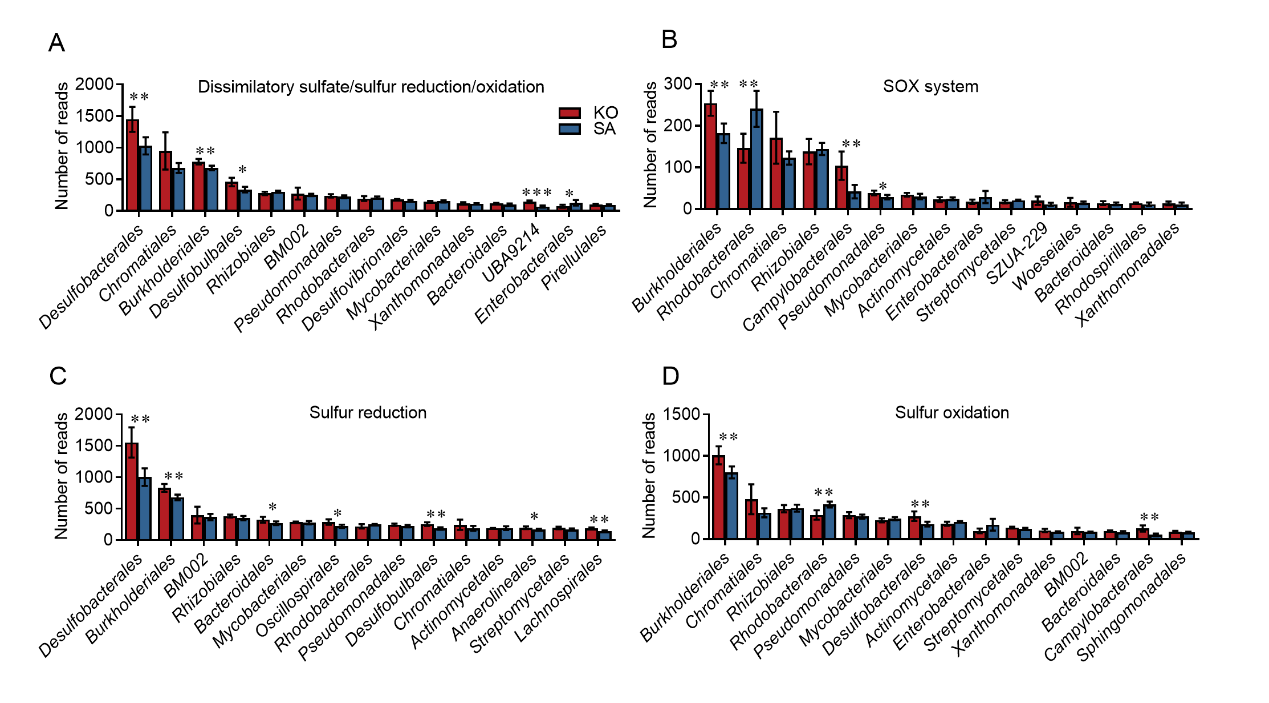


**Figure S9.** Relative abundances of top 15 abundant microbial taxa involved in dissimilatory sulfate/sulfur reduction/oxidation (A), SOX system (B), sulfur reduction (C) and sulfur oxidation (D). Bars represent the normalized number of reads for each order at a random subsampling of 8,370,184 sequences per sample. Data are presented as mean ± SD (standard deviation, n = 5). KO: *K. obovata*; SA: *S. apetala*.


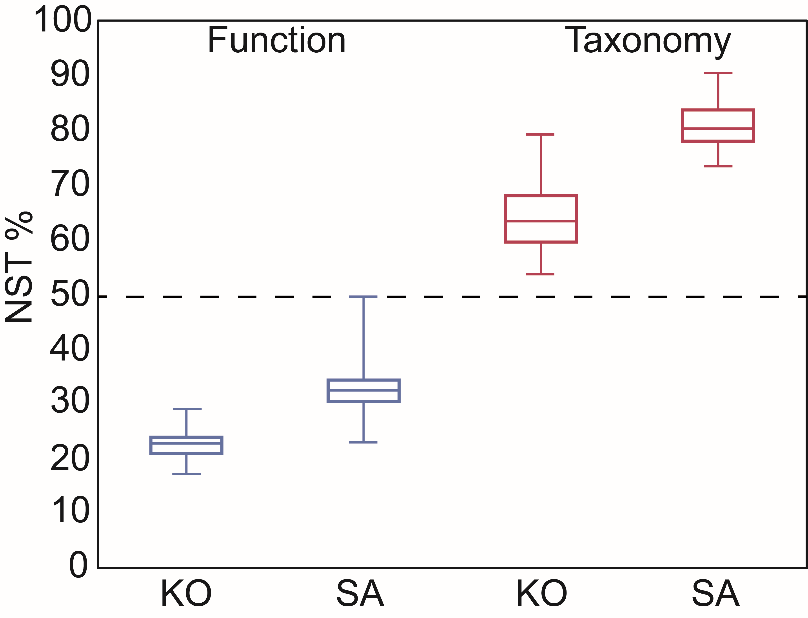


**Figure S10.** Assembly mechanisms of functional and taxonomical composition of rhizosphere microbiomes of *K. obovata* (KO) and *S. apetala* (SA). NST was calculated based on the Bray-Curtis methods.

**
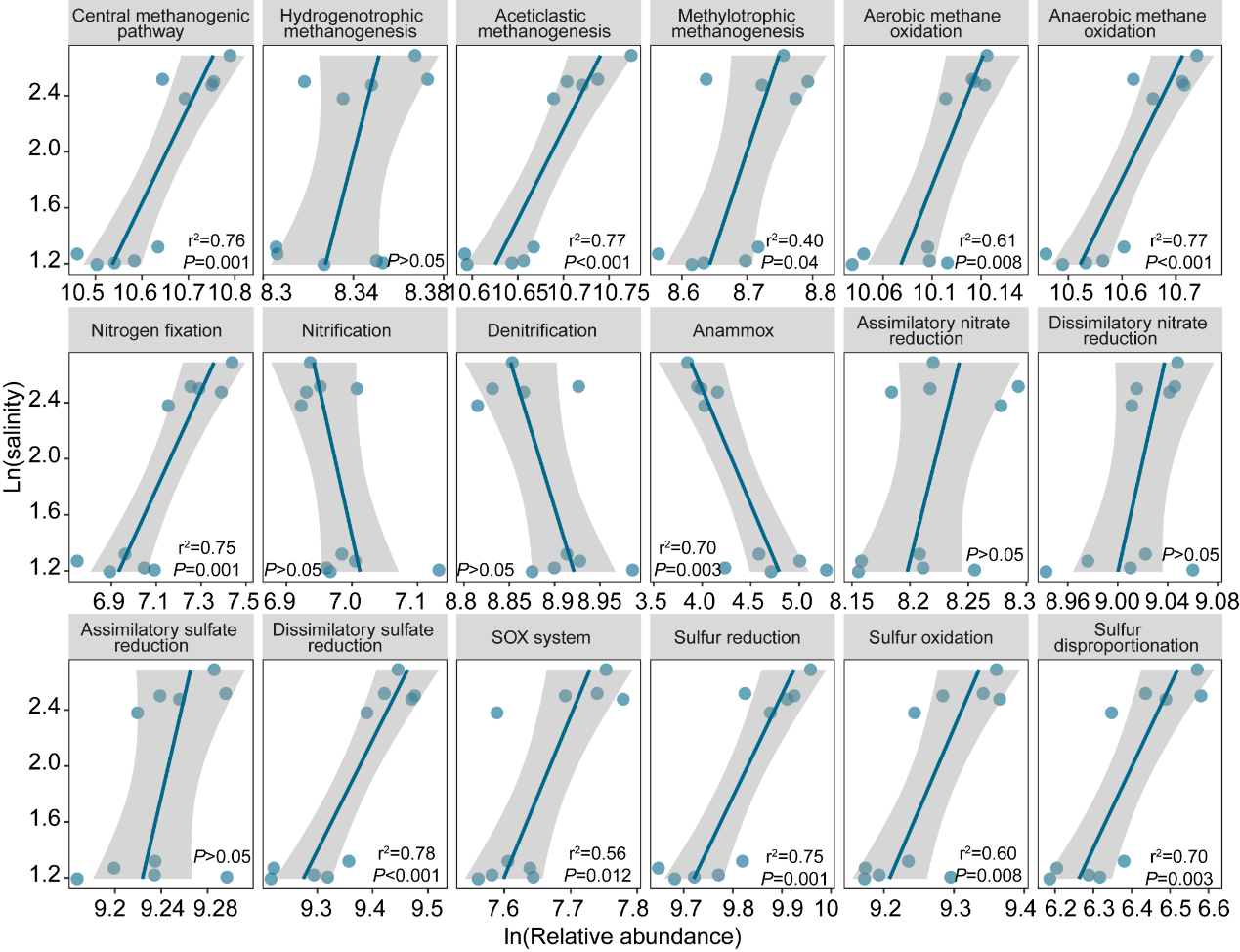
**

**Figure S11.** Linear regression analysis of correlations between salinity and relative abundances of CH_4_, N and S cycling pathways.

**
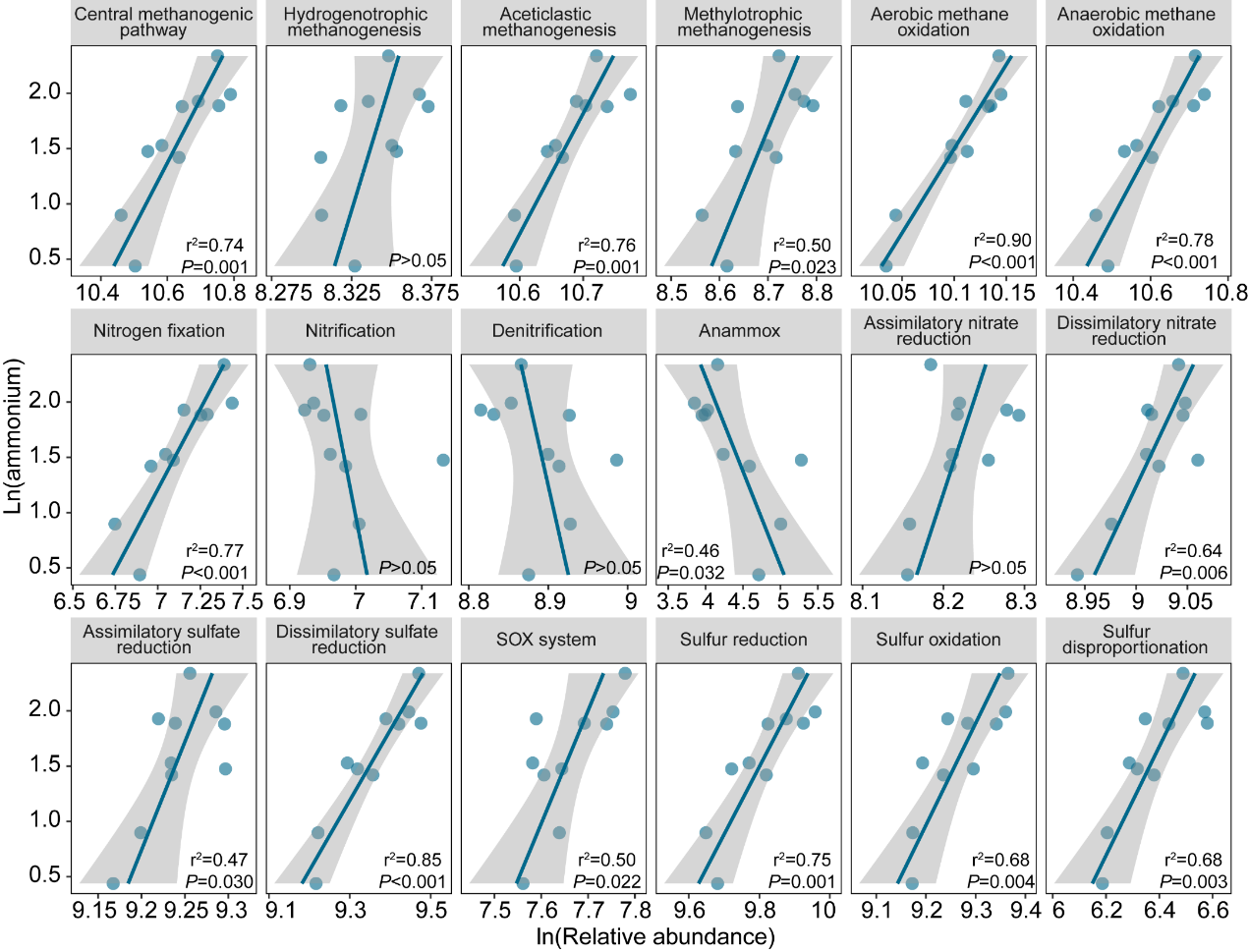
**

**Figure S12.** Linear regression analysis of correlations between ammonium and relative abundances of CH_4_, N and S cycling pathways.


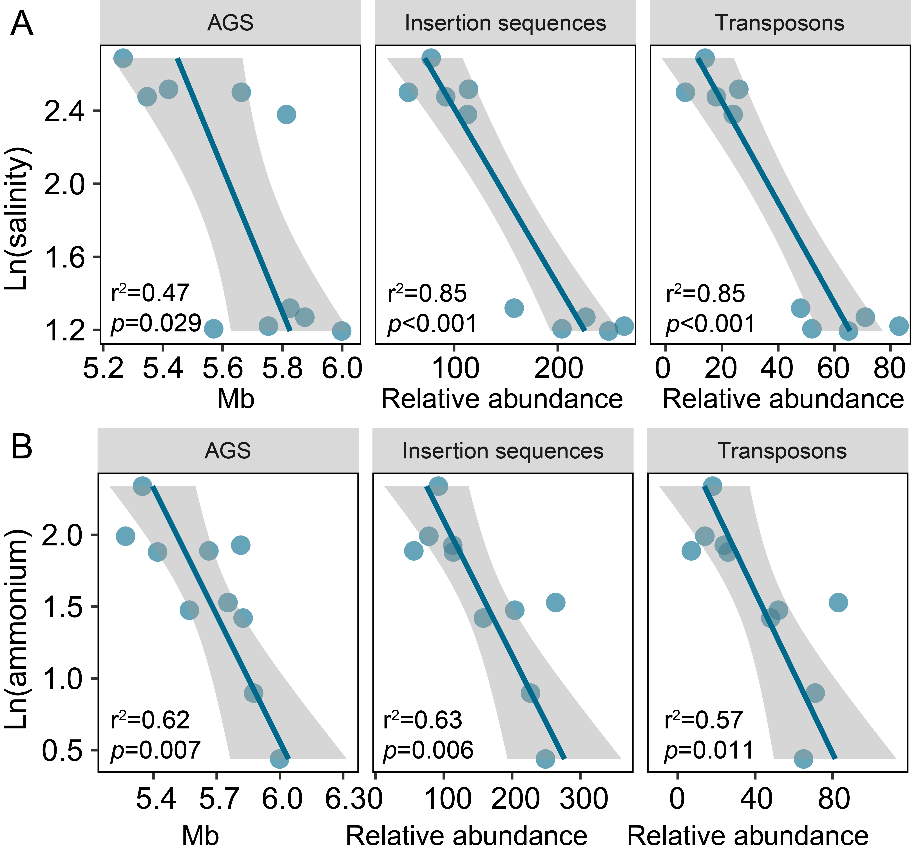


**Figure S13.** Linear regression analysis of correlations between salinity (A) and ammonium (B) with average genome size and relative abundances of insertion sequences and transposons.


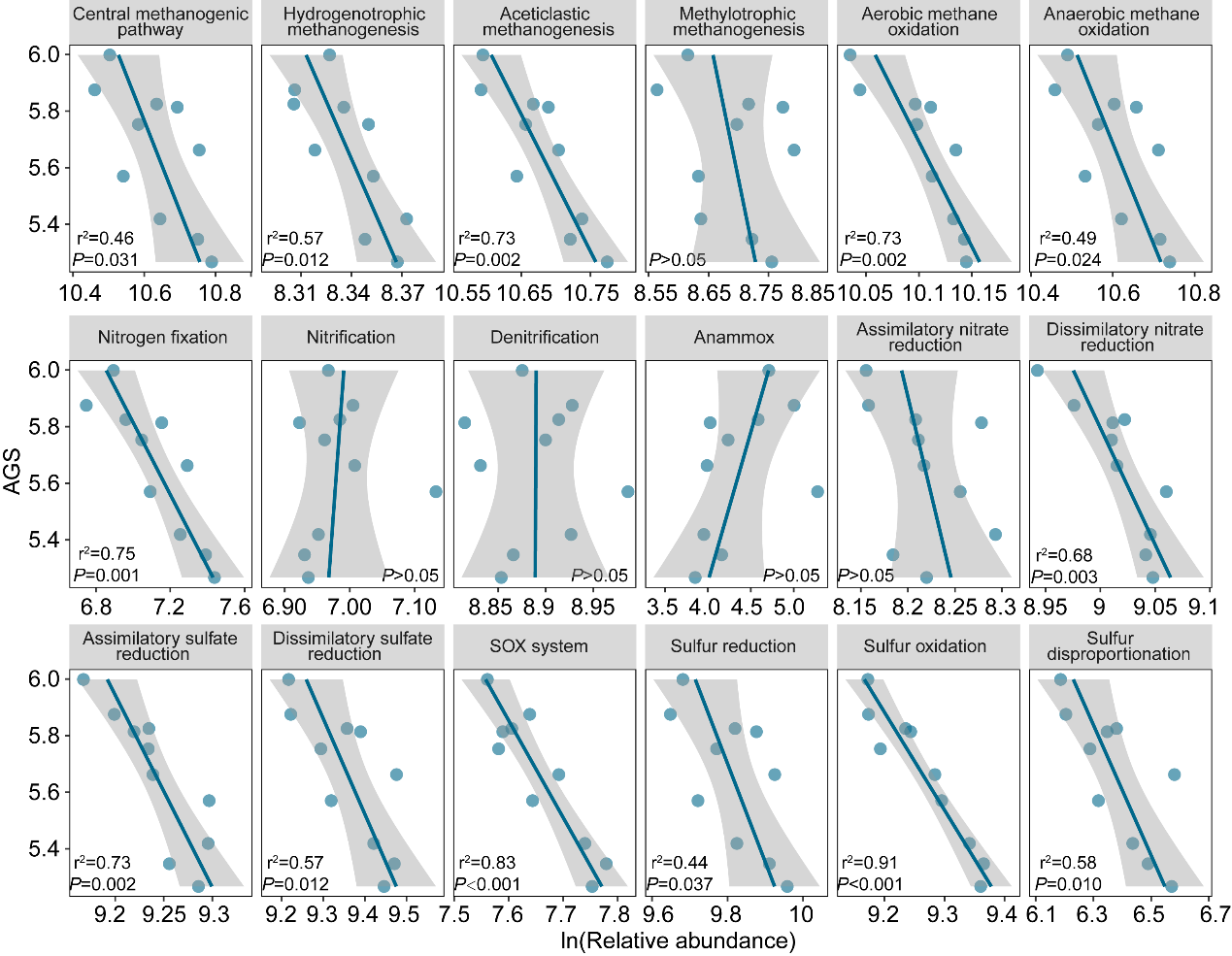


**Figure S14.** Linear regression analysis of correlations between average genome size and relative abundances of CH_4_, N and S cycling pathways.


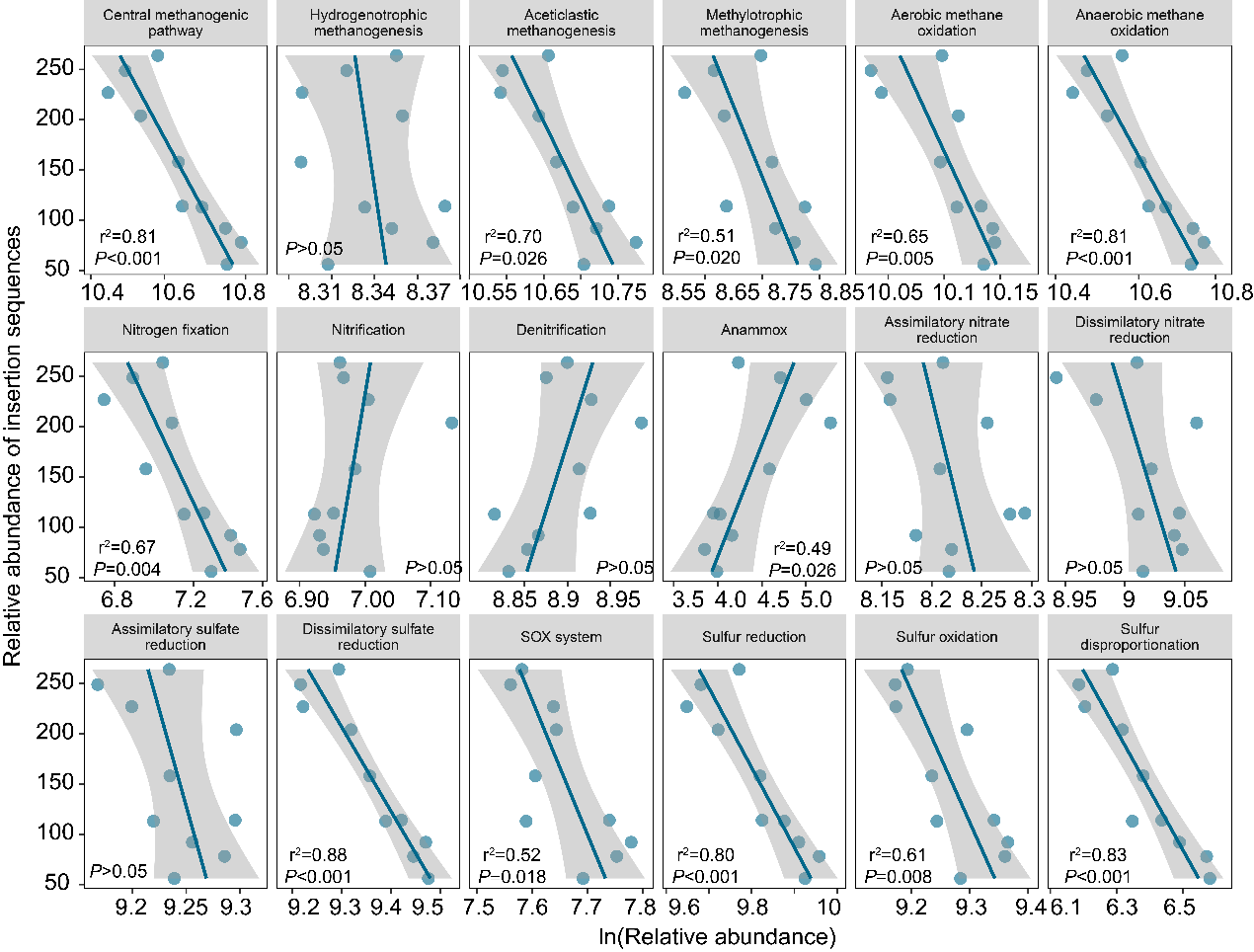


**Figure S15.** Linear regression analysis of correlations between relative abundances of insertion sequences and CH_4_, N and S cycling pathways.


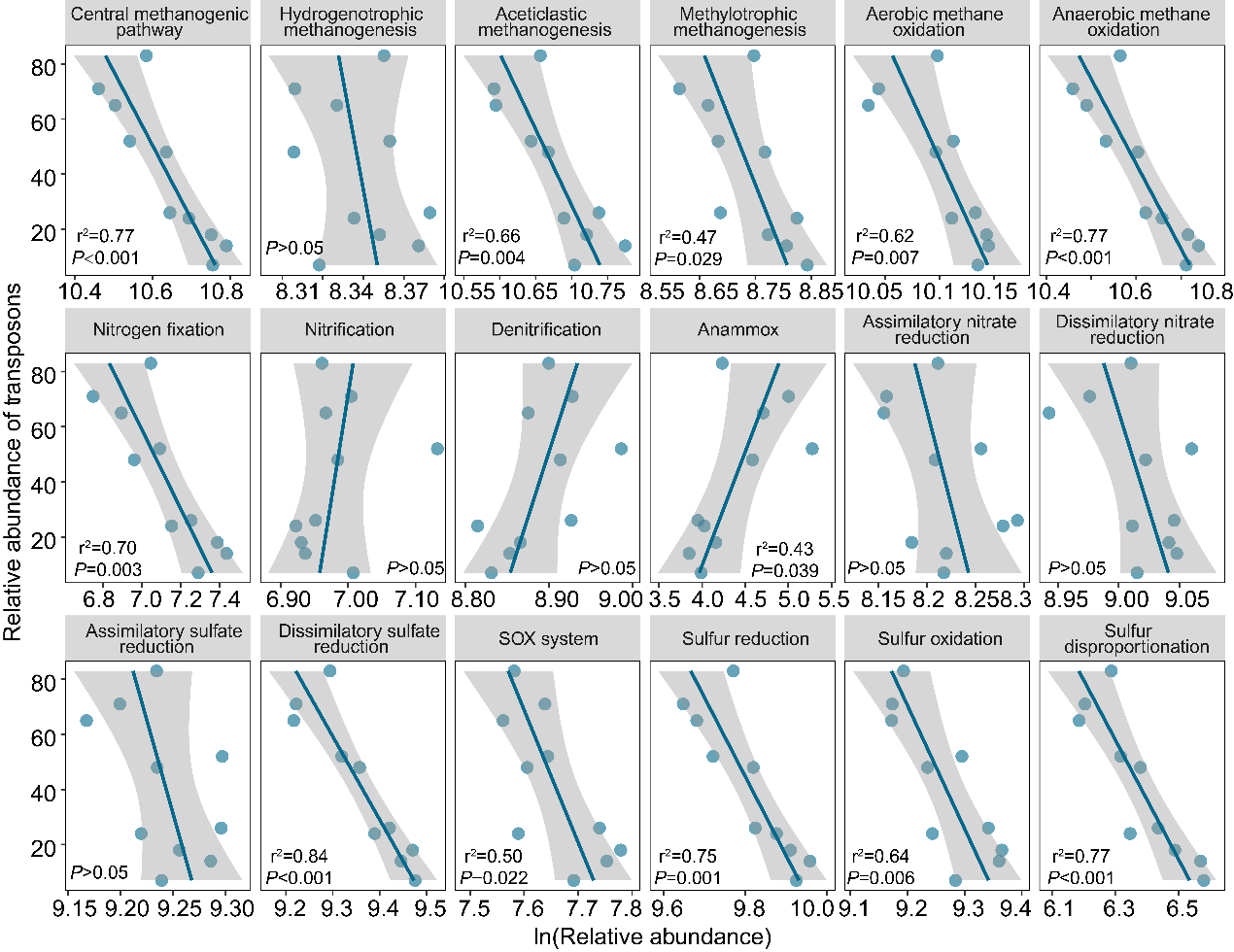


**Figure S16.** Linear regression analysis of correlations between relative abundances of transposons and CH_4_, N and S cycling pathways.
